# Mechanisms of amyloid-β34 generation indicate a pivotal role for BACE1 in amyloid homeostasis

**DOI:** 10.1038/s41598-023-28846-z

**Published:** 2023-02-07

**Authors:** Irem Ulku, Filip Liebsch, S. Can Akerman, Jana F. Schulz, Luka Kulic, Christoph Hock, Claus Pietrzik, Alessandro Di Spiezio, Gopal Thinakaran, Paul Saftig, Gerhard Multhaup

**Affiliations:** 1 Integrated Program in Neuroscience, McGill University, Montreal, QC H3G 0B1 Canada; 2 Department of Chemistry, Institute of Biochemistry, University of Cologne, 50674 Cologne, Germany; 3 Department of Pharmacology and Therapeutics, McGill University, Montreal, QC H3G 1Y6 Canada; 4 Institut Für Chemie Und Biochemie, Freie Universität Berlin, 14195 Berlin, Germany; 5 Roche Pharma Research & Early Development, F.Hoffmann-La Roche Ltd., 4070 Basel, Switzerland; 6 Institute for Regenerative Medicine, Un Iversity of Zurich, 8952 Schlieren, Switzerland; 7 Neurimmune AG, 8952 Schlieren, Switzerland; 8 Department Molecular Neurodegeneration, Institute of Pathobiochemistry, University Medical Center of the Johannes Gutenberg-University of Mainz, Duesbergweg 6, 55099 Mainz, Germany; 9 Biochemisches Institut, CAU Kiel, Olshausenstr. 40, 24098 Kiel, Germany; 10 Department of Molecular Medicine and Byrd Alzheimer’s Institute, University of South Florida, Tampa, FL 33613 USA; 11 Present Address: Cardiovascular and Metabolic Sciences, Max Delbrück Center for Molecular Medicine in the Helmholtz Association (MDC), 13125 Berlin, Germany

**Keywords:** Biochemistry, Biological techniques, Cell biology, Molecular biology, Neuroscience, Diseases, Medical research

## Abstract

The beta‑site amyloid precursor protein (APP) cleaving enzyme (BACE1) was discovered due to its “amyloidogenic” activity which contributes to the production of amyloid-beta (Aβ) peptides. However, BACE1 also possesses an “amyloidolytic” activity, whereby it degrades longer Aβ peptides into a non‑toxic Aβ34 intermediate. Here, we examine conditions that shift the equilibrium between BACE1 amyloidogenic and amyloidolytic activities by altering BACE1/APP ratios. In Alzheimer disease brain tissue, we found an association between elevated levels of BACE1 and Aβ34. In mice, the deletion of one BACE1 gene copy reduced BACE1 amyloidolytic activity by ~ 50%. In cells, a stepwise increase of BACE1 but not APP expression promoted amyloidolytic cleavage resulting in dose-dependently increased Aβ34 levels. At the cellular level, a mislocalization of surplus BACE1 caused a reduction in Aβ34 levels. To align the role of γ-secretase in this pathway, we silenced Presenilin (PS) expression and identified PS2-γ-secretase as the main γ-secretase that generates Aβ40 and Aβ42 peptides serving as substrates for BACE1’s amyloidolytic cleavage to generate Aβ34.

## Introduction

Alzheimer disease (AD) is a progressive neurological disease characterized by intracellular neurofibrillary tangles and extracellular amyloid plaques, which are mainly composed of amyloid beta (Aβ) peptides^1,2^. Traditionally, AD research has focused on Aβ production and the role of secretases in Aβ generation. In general, the process is initiated when β-secretase (BACE1) cleaves the amyloid precursor protein (APP) to generate sAPPβ and APP-C99 and involves a second protease, namely γ-secretase processing APP-C99^3–7^. BACE1 is a type-I transmembrane aspartic acid protease^8^ whose optimal activity requires an acidic environment in endosomes and lysosomes^9,10^. The γ-secretase, which further cleaves APP-C99 into Aβ peptides of varying lengths (e.g., Aβ40 and Aβ42), exists as a complex with four subunits, including the catalytic subunit Presenilin-1 or 2 (PS1 or PS2)^11–14^. Although both PS1- and PS2-γ-secretases possess overlapping enzymatic properties, due to their distinct localization, they have different access to substrates and differently influence Aβ abundance. PS1 selectively recognizes substrates on the cell surface, whereas PS2 preferentially cleaves substrates in late endosomes and lysosomes^15^.

BACE1 has a relatively loose sequence specificity, and regions outside of its main cleavage site are less important for substrate selection^16^. This finding may explain why, in addition to its role in Aβ production (i.e., BACE1 amyloidogenic activity), BACE1 was found to cleave longer Aβ isoforms (e.g., Aβ40 and Aβ42) at position 34, i.e., the β34-site, which is a third BACE1 cleavage site in addition to the two canonical β- and the β’-sites^17,18^. However, the cut at the β34-site occurs only with longer Aβ peptides (such as Aβ40 and Aβ42) as substrates previously released from γ-secretase complexes^18^. Unlike other Aβ species, Aβ34 has been described as non-toxic and non-aggregating^19^; therefore, the β34-cleavage is due to an amyloidolytic BACE1 activity as opposed to an amyloidogenic activity, which initiates production of aggregation prone Aβ peptides.

More recently, Aβ34 has been discovered by us as an early biomarker of amyloid clearance activity in prodromal AD^20^. The Aβ34/Aβ42 ratio showed a better diagnostic accuracy for prodromal AD than the traditional Aβ40/Aβ42 ratio. CSF Aβ34 levels were elevated in early clinical stages of AD and correlated with Aβ clearance rates in subjects with evidence of cerebral amyloid deposition^20^. Analyses with cultured human primary pericytes that normally regulate the blood–brain barrier function revealed a time and dose dependent production of Aβ34 upon treatment with recombinant Aβ40 peptides^21^.

Numerous studies tried to correlate BACE1 activity with amyloid peptide production that resulted in conflicting findings both in vivo and in vitro. In transgenic mice overexpressing human BACE1, high BACE1 overexpression inhibited amyloid formation despite increased β-cleavage of APP^22^ which is in sharp contrast to the expectation that increased BACE1 activity is causing increased amyloid production. The same apparently paradoxical behavior was observed in several pharmacological studies^23,24^. Post-mitotic human neurons treated with low concentrations of BACE1 inhibitors resulted in the expected decreased cellular BACE1 activity but unexpectedly higher levels of longer Aβ forms^24^. These findings have shifted our interest to the amyloidolytic activity of BACE1 as a possible explanation which is a fairly neglected enzymatic function in the equation of production and elimination of Aβ peptides.

Here, we unraveled molecular and cellular aspects of amyloidogenic and amyloidolytic activities of BACE1. We provide in vivo and in vitro evidence that the BACE1/APP ratio primarily determines BACE1-mediated Aβ clearance: In AD brain tissue, we found levels of both BACE1 and Aβ34 approximately two-fold elevated. Analysis of brain cortices from wild-type mice and mouse lines with reduced expression levels of BACE1 revealed an association between BACE1 and Aβ34. Impaired BACE1 localization to the endo-lysosomal system primarily affected Aβ34 levels. A decrease in Aβ34 levels observed upon downregulation of PS2, while Aβ40 and Aβ42 levels remained unaltered, implies a role for PS2 in Aβ34 generation in the endo-lysosomal system in conjunction with BACE1. Thus, our data provide molecular explanations for the previously reported paradoxical inverse relationship of low BACE1 expression and high Aβ levels and vice versa*.*

## Results

### BACE1’s amyloidogenic and amyloidolytic in vivo activities are determined by the enzyme to substrate ratio

**Figure 1:**
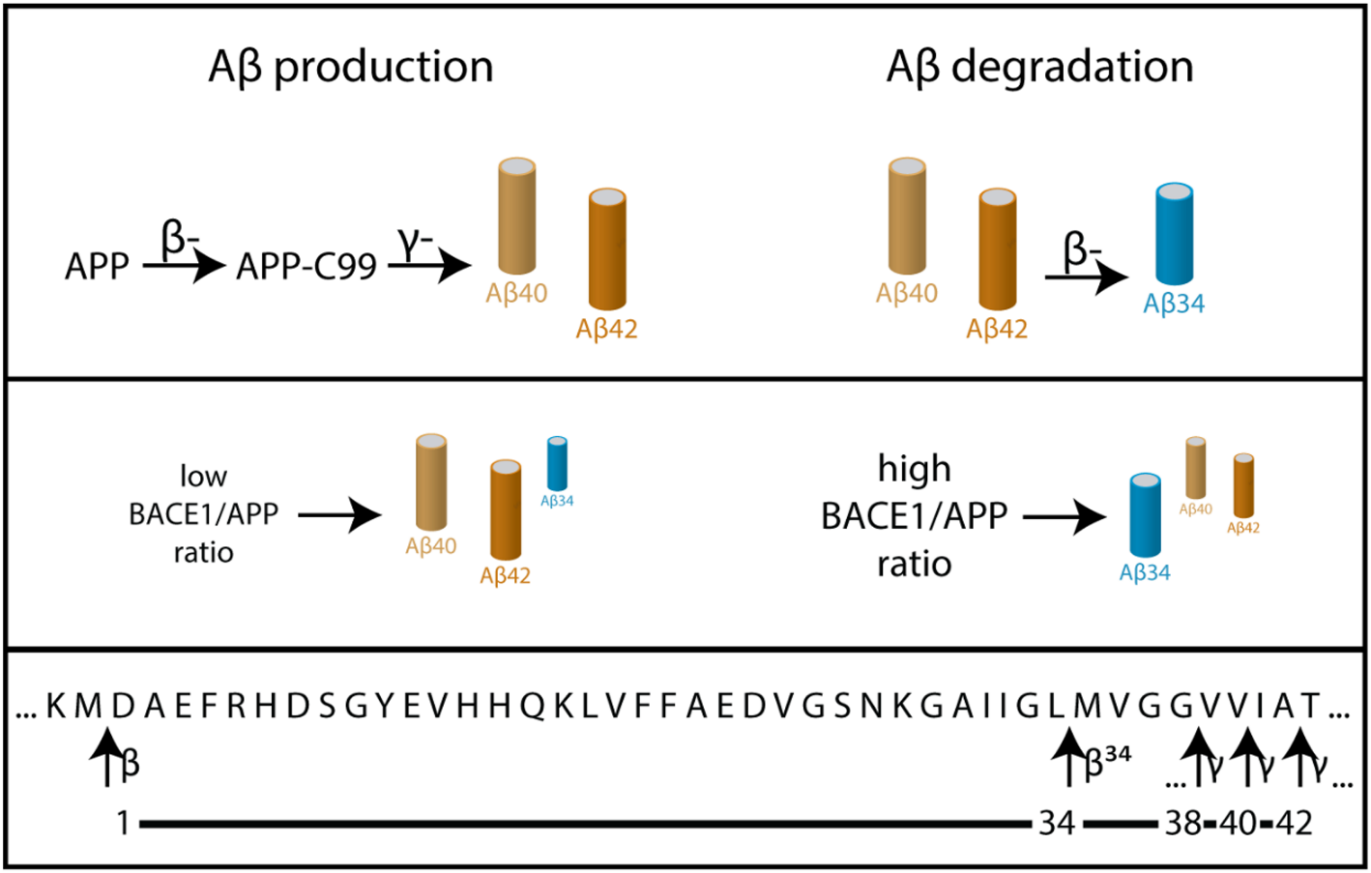
APP processing by β- and γ-secretases and amyloid degradation into Aβ34 at low and high BACE1/APP ratios. In the amyloidogenic pathway, sequential cleavage of APP by β-secretase and γ-secretase generates Aβ species of varying lengths including Aβ38, Aβ40 and Aβ42. In the Aβ amyloidolytic pathway, Aβ peptides resulting from the production pathway can be cleaved by β-secretase at the β34 site as part of the degradation pathway yielding the C-terminally truncated Aβ species, Aβ34. Under low BACE1/APP ratio, Aβ40 and Aβ42 levels are higher than Aβ34. In contrast, under high BACE1/APP ratio, Aβ34 levels increase as more Aβ40 and Aβ42 are degraded into Aβ34 by BACE1.

**Figure 2:**
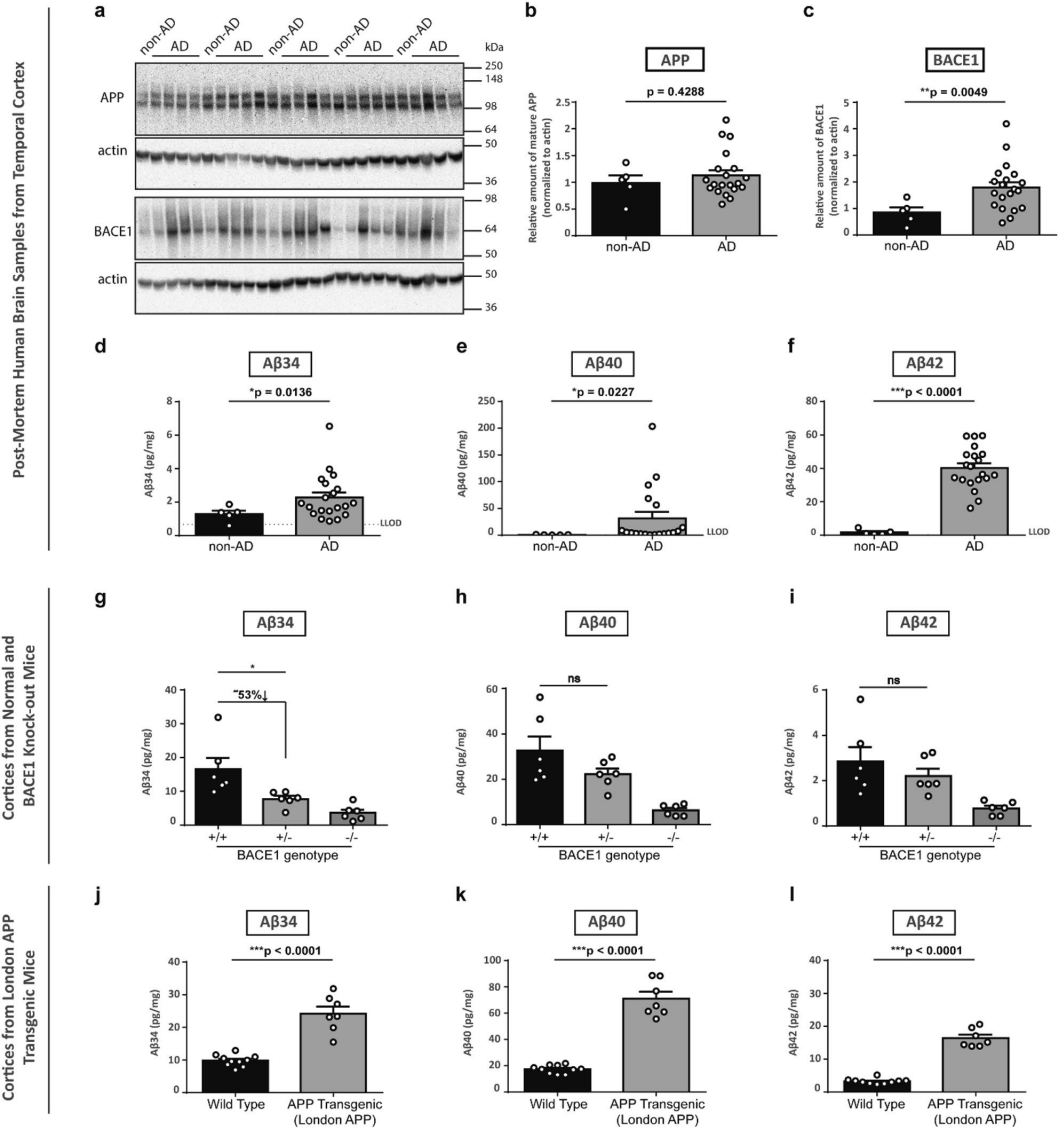
Aβ34 levels in AD post-mortem brain and in mouse brain tissue correlated with altered BACE1 expression and enhanced Aβ40 and Aβ42 levels. Expression of APP and BACE1, and Aβ34, Aβ40 and Aβ42 levels from post-mortem temporal brain and mouse cortices homogenates were analyzed by Western blot and MSD assays, respectively. Uncropped blots are included in a Supplementary Information file. Representative Western blot for the examination of APP and BACE1 expression levels (**a**). Quantification of relative protein amounts of APP (**b**) and BACE1 (**c**) of AD and non-AD. Absolute amounts of Aβ34 (**d**, **g**, and **j**), Aβ40 (**e**, **h**, and **k**) and Aβ42 (**f**, **i**, and **l**) determined with MSD 4-plex assays. For BACE1 knockout mice, cortices of 6 months-old 3 females and 3 males (**g**–**i**) and for London APP Transgenic mice, cortices of 6 months-old 7 females and 3 males (for WT) and 4 females and 3 males (for transgenic) (**j**–**l**) were analyzed. Data (**b**–**f**) were analyzed using unpaired Welch’s t-tests (due to violations of the normality assumption). Bars and error bars indicate mean ± s.e.m. (**b**) t(7) = 0.84, (**c**) t(13) = 3.34, (**d**) t(20) = 2.71, (**e**) t(18) = 2.54, (**f**) t(21) = 13.42. Data (**d**–**f** and **j**–**l**) were analyzed by unpaired t-test. Data (**g**–**i**) were analyzed by 1-WAY ANOVA and Tukey’s post-hoc tests were performed for pairwise comparisons; selected comparisons are highlighted ***p < 0.001, **p < 0.01, *p < 0.05. (**g**) Aβ34, 1-WAY ANOVA, F(2,15) = 10.33, p = 0.0015, (**h**) Aβ40, 1-WAY ANOVA, F(2,15) = 11.75, p = 0.0009, (**i**) Aβ42, 1-WAY ANOVA, F(2,15) = 6.637, p = 0.0086.

To test whether there is a dichotomy between the amyloidogenic and amyloidolytic roles of BACE1 (Fig. 1) in vivo, we measured cortical Aβ levels in human brain tissue, in wild-type, BACE1 knock-out (BACE1 −/−), heterozygous mice with half of the normal amount of active BACE1 (BACE1 +/−), and in APP transgenic mice expressing the human APP gene with the London mutation V717I. First, we examined BACE1 and APP levels in post-mortem human temporal cortical samples from 20 AD patients and 5 controls (Fig. 2a-c; Supplemental Table 1). Western blot analysis revealed that cerebral BACE1 levels were ~ 2.1-fold elevated in AD patients compared to non-AD (Fig. 2c), which is in agreement with previous studies where BACE1 protein and activity levels were found to be increased in the brain regions affected by amyloid deposition^25–28^. Cerebral APP levels did not differ between AD patients and non-demented controls (Fig. 2b).

We hypothesized that a surplus of BACE1 would lead to increased Aβ34, given that BACE1 levels are significantly elevated in AD, while APP levels and Aβ40 and Aβ42 production rates do not change^29^. Therefore, levels of Aβ34 and the longer Aβ species, i.e., Aβ40 and Aβ42 resulting from the classical amyloidogenic processing of APP, were measured in human brain extracts using our previously developed 4-plex assay (MSD—Meso Scale Discovery)^20^. Aβ34 levels were elevated ~ 1.8 fold, which is very similar to the ~ 2.1-fold elevated BACE1 level in AD brain tissue. Thus, both cerebral BACE1 and Aβ34 levels increased approximately ~ twofold (Fig. 2d), suggesting that excess BACE1 may generate more Aβ34 in AD brain tissue. A Spearman test suggests an overall trend for the correlation (ρ = 0.3575, p = 0.0795) between relative BACE1 values and absolute Aβ34 levels. Notably, in agreement with this data, serum BACE1 activity was found to be ~ 30% higher in AD patients compared to controls^30,31^. In addition, Aβ40 and Aβ42 species were significantly elevated in the AD group by ~ 44- and ~ 23-fold, respectively (Fig. 2e and f), possibly due to aggregated amyloid as previously reported^32^.

To test whether the absence of aggregated amyloid yields a similar relationship between BACE1 and the different Aβ species, we measured Aβ34, Aβ40 and Aβ42 levels in the cortices of 6 months-old wild-type (+/+), heterozygous (+/−) and homozygous BACE1 knockout (−/−) mice (3 females and 3 males for each genotype) expressing endogenous levels of APP. We observed that Aβ34 levels were significantly reduced in BACE1 +/− animals but not the Aβ40 and Aβ42 levels. The loss of one BACE1 allele led to a significant decrease in Aβ34 levels (compare BACE1 +/+ and BACE1 +/−) (Fig. 2g), while no significant effects were observed for Aβ40 and Aβ42 (Fig. 2h and i). Unaltered levels of Aβ40 and Aβ42 were also observed by others under the condition of lowered endogenous BACE1 activity^20,33–35^. Thus, Aβ34 levels positively correlate with BACE1 levels, which is not the case for Aβ40 and Aβ42 levels that remain unaltered with the loss of one BACE1 allele.

Then, we utilized transgenic mice expressing human APP, i.e., in vivo overexpressing conditions, to analyze the effect of substrate overexpression. Cortical Aβ levels of wild-type animals (7 females and 3 males) were compared to cortical Aβ levels of 6 months-old (pre-plaque) mice with the London mutation driven by the Thy1 promoter (4 females and 3 males). Aβ34 levels were increased ~ 2.5 fold and Aβ40 and Aβ42 were found elevated ~ four- and ~ fivefold, respectively (Fig. 2j-l). Western blot analysis revealed that APP transgenic mice had ~ 2.2 fold more APP and showed normal levels of BACE1 (Supplementary Fig. 1). Altogether, the results show that amyloidogenic activity was maintained with a single copy of the endogenous BACE1 gene (Fig. 2h and i), while amyloidolytic activity was reduced upon the loss of one BACE1 gene copy (Fig. 2g).

### BACE1 expression promotes Aβ34 generation from APP and APP-C99 in vitro

To further determine how increased APP or BACE1 expression is influencing the balance between amyloidogenic and amyloidolytic cleavages, we tested cells transfected with increasing amounts of cDNA of either BACE1 or APP.

**Figure 3:**
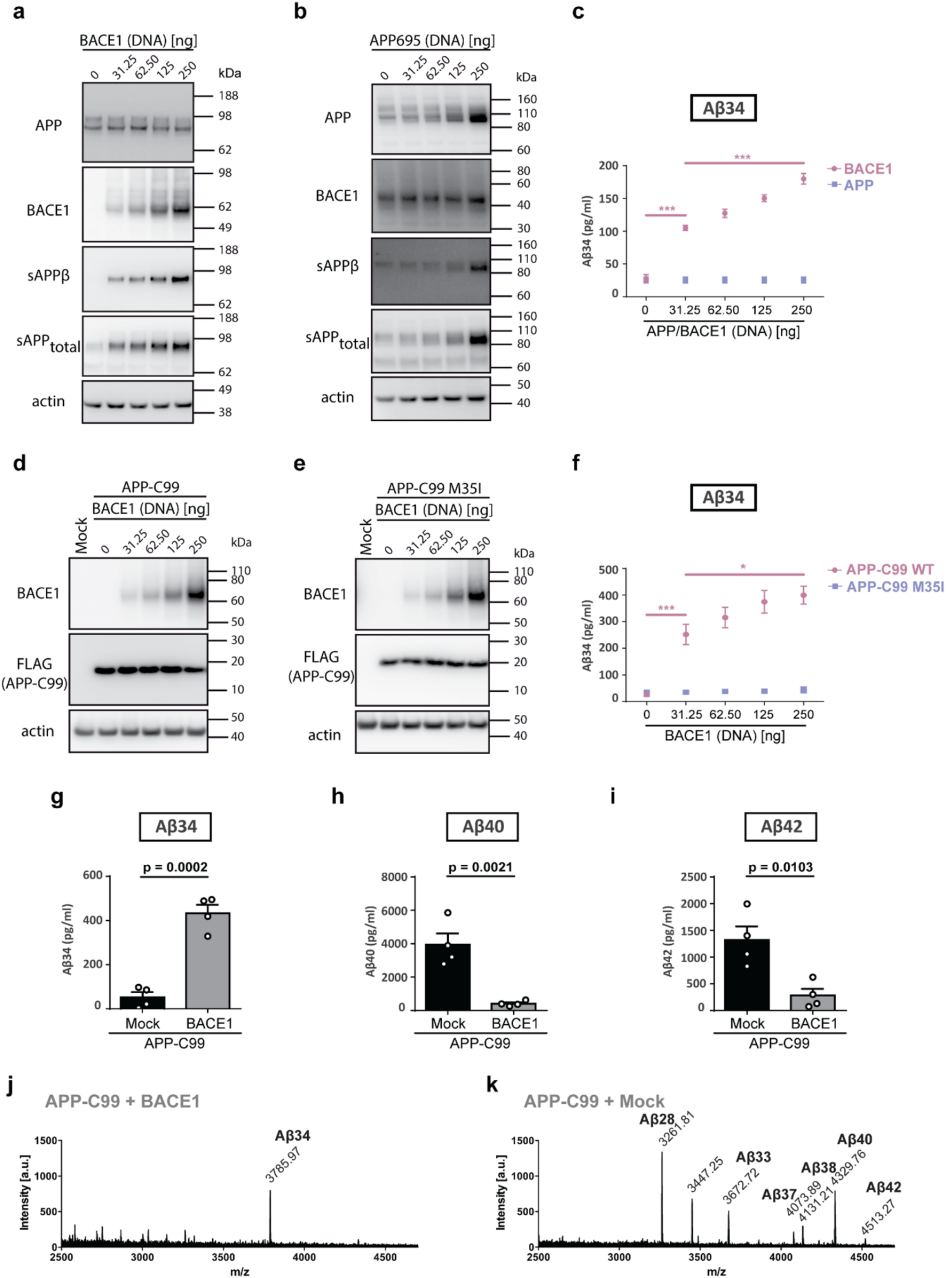
BACE1 overexpression and co-expression with APP-C99 enhanced Aβ34 production from Aβ40 and Aβ42. Expression of APP, APP-C99, and BACE1 and Aβ34 generated from endogenous levels of APP and under APP and APP-C99 overexpression conditions (wild-type APP-C99 and APP-C99 M35I mutant) were analyzed by Western blot and ELISA, respectively. Uncropped blots are included in a Supplementary Information file. HEK293T cells were transfected with indicated increasing amounts of cDNA coding for BACE1 (**a**) or APP695 (**b**) or APP-C99 and BACE1 (**d**) or APP-C99 M35I and BACE1 (**e**). Representative Western blots from 5 independent experiments for the examination of APP, BACE1, sAPPβ and sAPP_total_ expression (**a**, **b**, **d** and **e**). Quantification of absolute amounts of Aβ34 by ELISA (**c** and **f**). Aβ generation from BACE1 and/or APP-C99 overexpressing HEK293T cells was analyzed by ELISA, and immunoprecipitation (IP) Matrix Assisted Laser Desorption/Ionization (MALDI) mass spectrometry (MS). Cells were transfected with APP-C99, BACE1, and/or empty vector (Mock). Quantification of absolute amounts of Aβ34 (**g**), Aβ40 (**h**), and Aβ42 (**i**) with specific ELISAs. Aβ species were immunoprecipitated with monoclonal W02 and analyzed by MALDI-MS. Representative spectra from 3 independent experiments (**j** and **k**). Bars and error bars indicate mean ± s.e.m. Tukey’s post-hoc tests were performed for pairwise comparisons; selected comparisons are highlighted ***p < 0.001, *p < 0.05. (**c**) Aβ34, 1-WAY ANOVA, F(4,20) = 89.90, p < 0.0001, (**f**) Aβ34, 1-WAY ANOVA, F(5,24) = 28.28, p < 0.0001, (**g**) t-test, t(6) = 8.44, (**h**) t-test, t(6) = 5.16, (**i**) t-test, t(6) = 3.68. Linear regression was performed for the linearity test between BACE1 overexpression and Aβ34 levels (between 31.25 ng and 250 ng BACE1 DNA treatment). F(4,20) = 72.21, p < 0.0001 with the equation y = 0.3225x + 103.0.

When corresponding Western blots were quantified, increases in sAPPβ and sAPP_total_ were observed under both APP695 and BACE1 overexpression conditions. However, the increase in APP processing by β-secretase, indicated by sAPPβ levels, was more pronounced under BACE1 overexpression compared to APP695 overexpression (Supplementary Fig. 2a-d). Upon dose-dependently increased BACE1 levels (Fig. 3a), Aβ34 levels started to rise above Lower Limit of Detection (LLOD) with the lowest amount of BACE1 transfected and in a linear manner (y = 0.3225x + 103.0, p < 0.0001) over the entire range (Fig. 3c). APP overexpression in HEK293T cells resulted in increased levels of APP but left Aβ34 levels unaltered (Fig. 3b and c). These results demonstrate that a surplus of BACE1, but not of APP, promotes amyloidolytic cleavage yielding higher Aβ34 levels in non-neuronal cells where endogenous BACE1 expression is naturally low^36^.

To study Aβ34 formation independently from prior β-secretase cleavage of APP by BACE1 (cleavage at the Asp^1^ residue), we used a construct that encodes for the immediate γ-secretase substrate β-CTF, termed as APP-C99^37,38^ (Fig. 3d). HEK293T cells were co-transfected with both increasing concentrations of BACE1 and a constant amount of APP-C99 and expression was verified by Western blot (Fig. 3d and e). Aβ34 levels were below LLOD under mock condition. A steady rise of Aβ34 levels was observed in BACE1 and APP-C99 co-transfected cells. Additionally, Aβ40 and Aβ42 levels were dose-dependently reduced in these co-transfected cells (Supplementary Fig. 2e and f). To prove that Aβ34 generation depended on the cleavage at the β34 site, we used an engineered mutant construct, where amino acid residue 35 (M35) of APP-C99 was mutated to Ile encoding for APP-C99 M35I (Fig. 3e). Under these conditions amyloidolytic cleavage at the β34 site was abolished (Fig. 3f).

The quantitative analysis of the conditioned media from APP-C99 and BACE1 co-transfected HEK293T cells showed that BACE1 overexpression increased Aβ34 levels (Fig. 3g) while Aβ40 and Aβ42 levels were diminished (Fig. 3h and i). Surprisingly, Aβ34 peptides were the predominating species under BACE1 and APP-C99 co-expressing conditions, as verified in immunoprecipitates by Matrix-Assisted Laser Desorption/Ionization Mass Spectrometry (MALDI-MS) (Fig. 3j and k). Qualitative results confirm that longer and shorter Aβ species are released by cells only overexpressing APP-C99 (Fig. 3k) but increased BACE1 levels correspond with increased detection of Aβ34 species.

### Cellular localization of BACE1 modulates its amyloidolytic activity

Next, we verified whether Aβ34 is generated in the endo–lysosomal system. We tested BACE1 mutants with amino acid substitutions in the acidic cluster motif, DDISLL (residues 495–500 of BACE1 contained within its cytosolic C-terminal domain) that are well-known for altering intracellular localization and trafficking of BACE1. Notably, substitutions of D495 or L499-L500 in the [DE]XXXL[LI] signal^39^ were described to decrease endosomal localization and increase plasma membrane localization of BACE1^40,41^.

**Figure 4:**
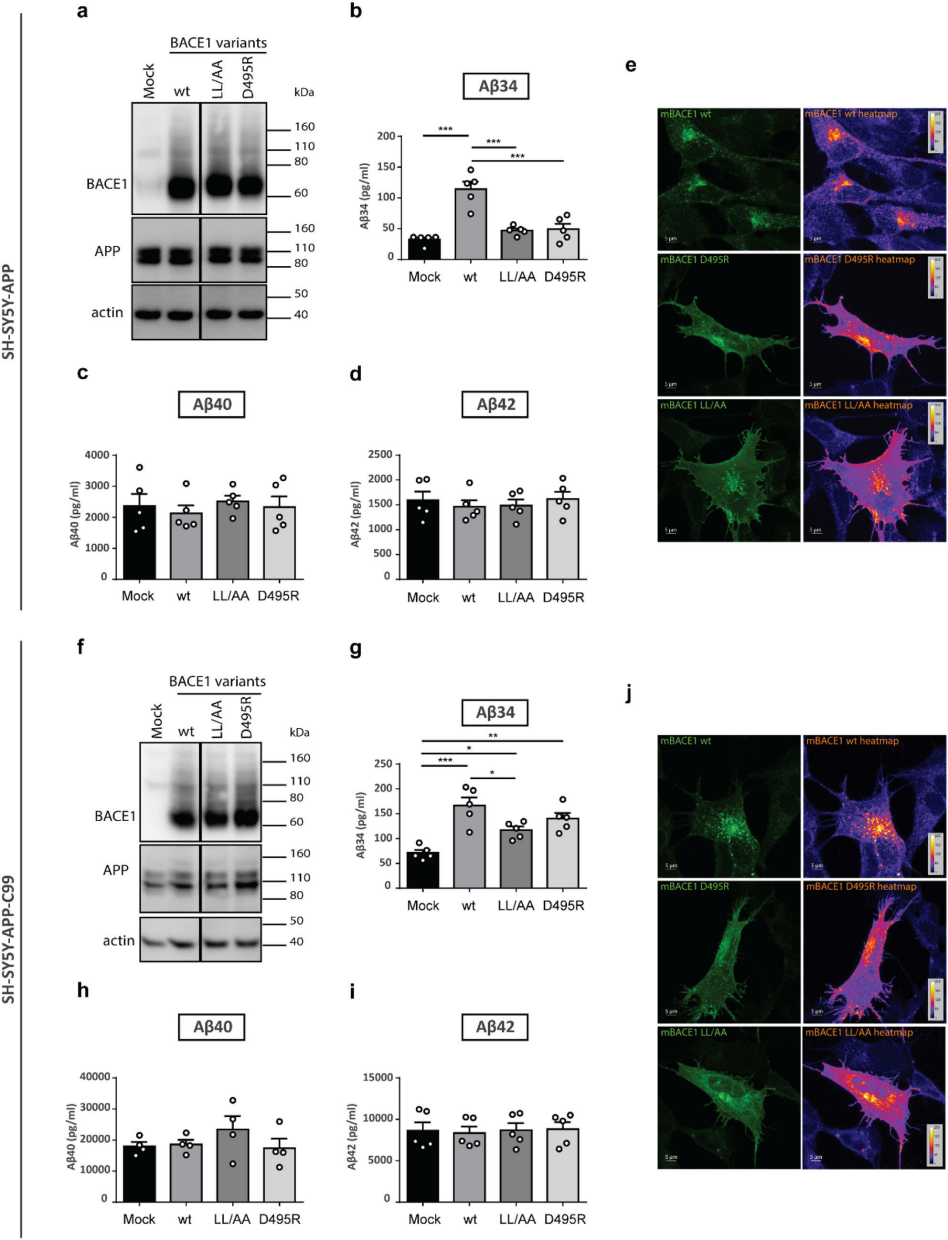
Altered localization of BACE1 to the endo-lysosomal system affected Aβ34 production. Expression of BACE1 mutants and Aβ34, Aβ40 and Aβ42 levels were analyzed by Western blot and ELISA assays, respectively. Uncropped blots are included in a Supplementary Information file. Localization of BACE1 encoded by mutant constructs was analyzed by ICC. Representative Western blots of BACE1 and APP expression from 5 independent experiments with SH-SY5Y-APP (**a**) and SH-SY5Y-APP-C99 cells (**f**) transfected with different variants affecting BACE1 trafficking or mock. Absolute amounts of Aβ34 (**b** and **g**), Aβ40 (**c** and **h**), and Aβ42 (**d** and **i**). Representative ICC heatmaps of BACE1 wild-type, D495R and LL/AA in SH-SY5Y-APP (**e**) and SH-SY5Y-APP-C99 (**j**) cells from 3 independent experiments. Bars and error bars indicate mean ± s.e.m. Tukey’s post-hoc tests were performed for pairwise comparisons; selected comparisons are highlighted ***p < 0.001, **p < 0.01, *p < 0.05. (**c**) Aβ34, 1-WAY ANOVA, F(3,16) = 21.41, p < 0.0001, (**d**) Aβ40, 1-WAY ANOVA, F(3,16) = 0.2724, p = 0.8444, (**e**) Aβ42, 1-WAY ANOVA, F(3,16) = 0.2775, p = 0.8408, (**g**) Aβ34, 1-WAY ANOVA, F(3,16) = 13.20, p < 0.0001, (**h**) Aβ40, 1-WAY ANOVA, F(3,16) = 0.9514, p = 0.4468, (**i**) Aβ42, 1-WAY ANOVA, F(3,16) = 0.05190, p = 0.9838.

We explored the amyloidolytic activity under the condition of impaired endosomal localization and trafficking using two different BACE1 constructs (LL/AA [DDIS*AA*] and D495R [*R*DISLL]) in cells either stably overexpressing full-length APP or APP-C99. Unlike Aβ40 and Aβ42 levels, which were approximately seven-and fivefold higher in APP-C99-overexpressing cells, respectively, compared to APP-overexpressing cells, Aβ34 levels were relatively similar in both cell types, which supports the results from APP-C99 and BACE1 co-transfected cells shown above (Fig. 2f) implying that BACE1 most likely is the limiting factor for amyloidolytic activity. At similar expression levels of wild-type BACE1 and of the mutant constructs (Fig. 4a and f), relative levels of Aβ40 and Aβ42 remained unaltered when compared to the control (“Mock”) (Fig. 4c, d, h and i). In contrast, Aβ34 levels were reduced by ~ 55% (compared to wt) for both BACE1 trafficking mutants in APP overexpressing cells (Fig. 4b) and by ~ 25% (LL/AA) and ~ 10% (D495R) in APP-C99 overexpressing cells (Fig. 4g). The observed effect was attenuated in APP-C99 overexpressing cells, likely due to an excessive supply of substrate, i.e., 10- (compare Fig. 4c and h) and sixfold higher levels (compare Fig. 4d and i) of Aβ40 and Aβ42, respectively.

We verified the cellular localization of BACE1 mutants that impair endosomal trafficking^40^ by immunocytochemistry (ICC). Briefly, wild-type BACE1 showed a punctate like staining (Fig. 4e and j) which overlapped with both the early-endosome marker EEA1 (early-endosome associated protein 1) and the lysosome marker LAMP1 (lysosome-associated membrane protein 1) in both cell types^42,43^ (Supplementary Fig. 3). Quantitative colocalization analyses showed a significantly reduced colocalization with early endosomal marker EEA1 and lysosomal marker LAMP1 for both BACE1 variants, LL/AA and D495R (Supplementary Fig. 3g and h), which is in agreement with previous reports^41,44,45^.

Altogether, quantification and colocalization results suggest that Aβ34 is mainly produced within the endo-lysosomal compartments, and mutations altering BACE1 localization impair Aβ34 production due to mislocalization or a delayed transport of the mutant enzyme.

### PS2 γ-secretase but not PS1 complexes contribute to Aβ34 production

Literature indicates that numerous C-terminally truncated Aβ species are generated by the γ-secretase complex in a PS1/2-dependent manner^46–48^ and that γ-secretase activity is required first to produce secreted Aβ species^17,47^ which are then cleaved again by BACE1 to generate Aβ34.

**Figure 5:**
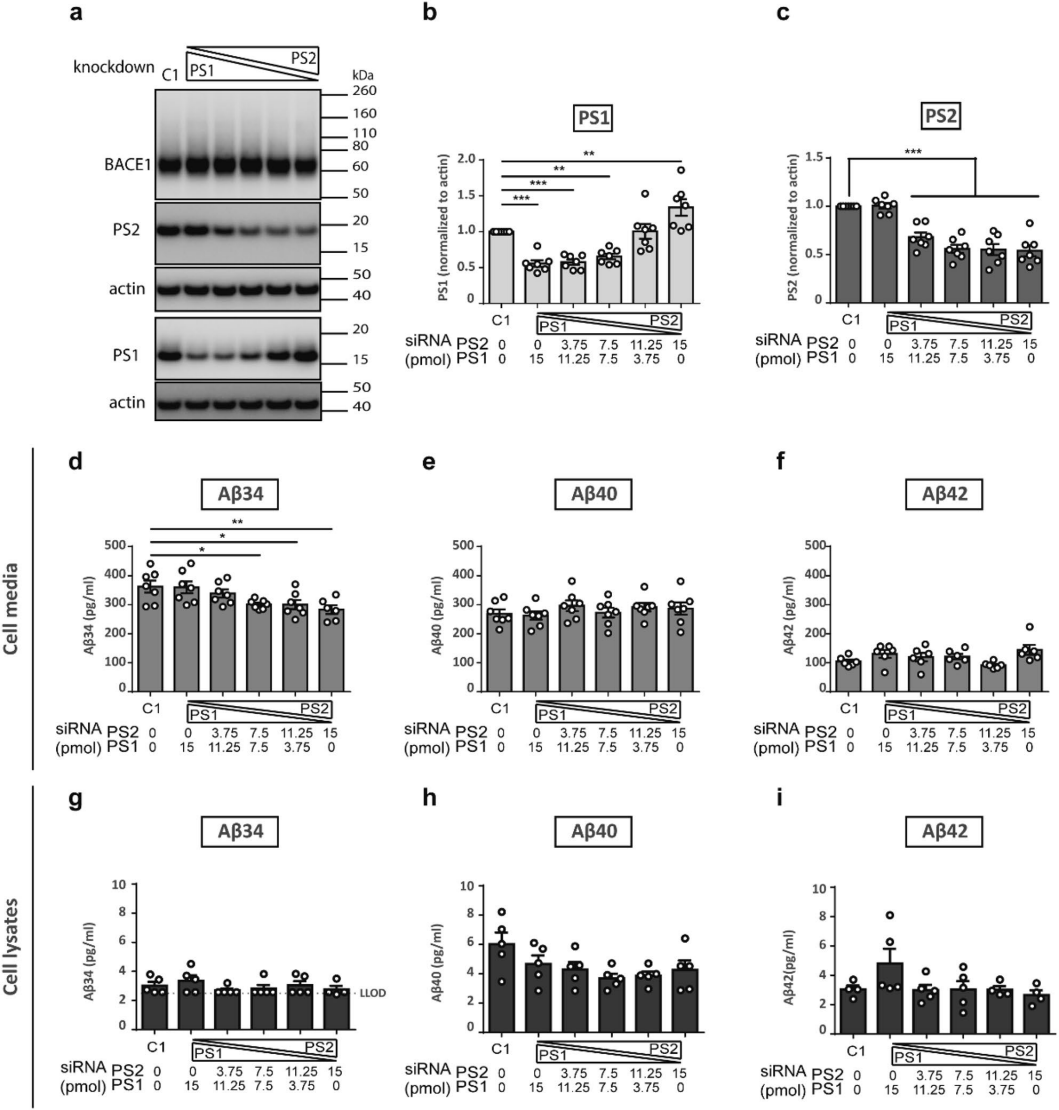
PS2 but not PS1 knockdown reduced Aβ34 levels from BACE1 overexpressing cells. Expression of PS1 and PS2 and Aβ levels were analyzed by Western blot, ELISA and MSD assays, respectively. Representative Western blots from 7 independent experiments for combinatorial PS1 and PS2 knockdown in SH-SY5Y BACE1 overexpressing cells (**a**). Uncropped blots are included in a Supplementary Information file. Quantification of relative amounts of PS1 (**b**) and of PS2 (**c**), and absolute amounts of Aβ34 (**d**), Aβ40 (**e**), and Aβ42 (**f**) in cell media by ELISA and Aβ34 (**g**), Aβ40 (**h**), and Aβ42 (**i**) in cell lysates by MSD. Bars and error bars indicate mean ± s.e.m. Dunnett’s post-hoc tests were performed for pairwise comparisons; selected comparisons are highlighted ***p < 0.001, **p < 0.01, *p < 0.05. (**b**) PS1, 1-WAY ANOVA, F(5,36) = 20.06, p < 0.0001, (**c**) PS2, 1-WAY ANOVA, F(5,36) = 26.37, p < 0.0001, (**d**) Aβ34, 1-WAY ANOVA, F(5,35) = 4.268, p < 0.005, (**e**) Aβ40, 1-WAY ANOVA, F(5,36) = 0.6677, p = 0.6504, (**f**) Aβ42, 1-WAY ANOVA, F(5,36) = 2.502, p = 0.0523, (**g**) Aβ34, 1-WAY ANOVA, F(5,23) = 0.8428, p = 0.5334, (**h**) Aβ40, 1-WAY ANOVA, F(5,24) = 2.276, p = 0.0791, (**i**) Aβ42, 1-WAY ANOVA, F(5,21) = 1.869, p = 0.1429.

To dissect the roles of PS1- and PS2-containing γ-secretase complexes in Aβ34 generation, we performed titration experiments with small interfering RNAs (siRNAs) to silence *PSEN1* or *PSEN2* expression. In a double knockdown titration experiment with either decreasing or increasing amounts of PS1 or PS2 siRNA and vice versa (Fig. 5), the total siRNA amount was equivalent to 15 pmol. The gradual downregulation of *PSEN1* or *PSEN2* was verified by Western blot analysis (Fig. 5a-c). Aβ34, Aβ40 and Aβ42 levels were quantified by ELISA in cell media (Fig. 5d-f) and by MSD in cell lysates (Fig. 5g-i). A significant gradual decrease in Aβ34 levels was uniquely observed with decreasing PS2 while Aβ40 and Aβ42 levels remained unchanged in cell media (Fig. 5d-f). The highest PS2 knockdown resulted in a significant reduction of Aβ34 by ~ 20% (Fig. 5d). In contrast, Aβ34, Aβ40 or Aβ42 levels remained constant in cell lysates (Fig. 5g-i). Notably, PS1 protein levels increased 1.5-fold above the levels yielded by controls upon gradual PS2 knockdown, likely as a compensatory reaction (Fig. 5b). This effect was specific for PS1 and not observed for PS2 since upon PS1 siRNA treatment, PS2 protein levels remained constant (Fig. 5c). Aβ34 levels were not affected by the compensatory increase of PS1 but surprisingly decreased with PS2 reduction. This result suggests that PS2-γ-secretase complexes possess a unique role in Aβ34 generation while PS1 is not involved.

Differential localization of PS1 and PS2 was verified by ICC analysis. Our results showed that PS1 is present throughout the cell while PS2 displayed punctate localization. PS2 co-localizes with both EEA1 and LAMP1 whereas PS1 does not (Supplementary Fig. 4). ICC quantification also showed a decrease in PS1 and PS2 expression upon PS1 and PS2 knockdown (15 pmol condition), respectively (Supplementary Fig. 5).

We verified the results of the combinatorial knockdown experiment with single knockdowns of either *PSEN1* or *PSEN2* (Supplementary Fig. 6). The gradual downregulation of *PSEN1* or *PSEN2* was analyzed by Western blot (Supplementary Fig. 6a–c and e–g) and Aβ34, Aβ40 and Aβ42 levels in cell media were quantified by ELISA (Supplementary Fig. 6d and i). Downregulation of *PSEN1* left Aβ34, Aβ40 and Aβ42 levels unaltered (Supplementary Fig. 6d) and PS2 levels did not change upon gradual PS1 knockdown as described above (Supplementary Fig. 6c). In agreement with data shown in Fig. 5b, PS1 levels showed an unexpected compensatory increasing trend upon gradual PS2 knockdown (Supplementary Fig. 6g). Similar to the combinatorial knockdown experiments (Fig. 5), a significant gradual decrease in Aβ34 levels was observed with decreasing PS2 while Aβ40 and Aβ42 levels remained unchanged (Supplementary Fig. 6h) and the highest PS2 knockdown resulted in an approximately 20% reduction of Aβ34 levels, confirming the result above that PS2-γ-secretase but not PS1 contributes to Aβ34 generation.

## Discussion

An imbalance between the formation and elimination of Aβ peptides has been suggested as the trigger in the pathogenesis of AD^49,50^. However, the knowledge about proteolytic degradation of Aβ discovered to date is rather limited to the family of amyloid-degrading enzymes (ADEs) with both membrane-bound and soluble members including extracellular matrix metalloproteinases (MMP2 and MMP9), IDE, NEP and ECE^19,51–54^.

Previous reports showed that a cleavage between L34 and M35 of the Aβ sequence exerted by BACE1 produced the non-amyloidogenic Aβ34 peptide, a soluble and non-toxic C-terminally truncated degradation product of longer Aβ peptides^17,18,55^. Aβ34 thus differs from aggregation prone Aβ species deposited in AD brain tissue. We identified Aβ34 as an indicator of amyloid clearance since Aβ34 was elevated in individuals with mild cognitive impairment^20^. Moreover, a significantly decreased Aβ34/Aβ40 ratio was observed in microvessels from AD patients due to a reduced proteolytic degradation of amyloid peptides in AD^21^.

Here, we provide mechanistic evidence in vitro and in vivo supporting a prominent role of BACE1 in Aβ clearance. Under conditions of either elevated levels of APP or of BACE1, Aβ34 production was only enhanced under a surplus of BACE1. Increasing amounts of BACE1 resulted in a dose-dependent increase in Aβ34 levels in all our experimental test systems. Specifically, the levels of Aβ34 depends directly on increased BACE1 levels in AD brain, i.e., Aβ34 levels were approximately twofold elevated in the brains of individuals with AD compared to non-demented controls and levels coincided with roughly twofold higher BACE1 levels in vivo*.* While increased BACE1 levels and its amyloidogenic activity in AD have been reported before^25–28^, the biological significance of BACE1 for amyloid clearance had remained enigmatic. We successfully confirmed an association between BACE1 expression and Aβ34 levels indicating amyloid clearance (i) in genetically modified mice where a single copy of the BACE1 gene (BACE1 +/−) halved cortical Aβ34 levels and (ii) in cell culture systems where the linearity between BACE1 and Aβ34 levels remained stable even at high BACE1/APP ratios. Thus, our findings provide an explanation for the previously reported and paradoxical inverse relationship for BACE1 expression and Aβ levels measured in in vitro and in vivo test systems under conditions of genetic and pharmacological manipulation of BACE1 expression^22,24,56–60^.

Further, we identified the endo-lysosomal system as the critical compartment for amyloidolytic cleavage of longer Aβ species into Aβ34 product. The finding that two BACE1 trafficking mutants known to impair endosomal trafficking^40^ reduced Aβ34 levels while Aβ40 and Aβ42 levels remained unchanged is in agreement with reports that BACE1 activity is optimal at acidic pH in early endosomes and lysosomes^40,61,62^. Further, knockdowns of either the PS1 or the PS2 subunit showed that Aβ34 levels were specifically reduced upon PS2 knockdown. Thus, PS2-γ-secretase, rather than PS1, is involved in Aβ34 generation which is in full alignment with their reported cellular activities, as PS2 selectively cleaves late endosomal/lysosomal localized substrates and generates the prominent pool of intracellular Aβ peptides^15^. This assumption implies that Aβ peptide substrates are originating from PS2-γ-secretase complexes for BACE1 amyloidolytic cleavage. In contrast to the present study, we previously overexpressed wild type and a loss-of-function variant of PS1 and proposed that BACE1-generated Aβ34 was dependent on PS1 activity^17^. Then, this effect came from overexpressed PS1 likely “mislocalized” to the endosomal system, and we did not observe this dependency upon knockdown of the endogenous protein in this study.

Thus, we propose that BACE1 amyloidolytic activity in the endo-lysosomal system might provide specificity and a spatial and temporal control of amyloid clearance through the BACE1-amyloidolytic-activity pathway. In agreement with this view, longer Aβ forms are more prone to aggregation in acidic compartments^63,64^ requiring that clearance of Aβ40 and Aβ42 in acidic compartments is essential and must be highly effective.

Thus, we here addressed the yet undefined role of BACE1 in amyloid degradation*,* explaining why increased BACE1 activity is not leading to increased amyloid production but decreased Aβ40 and Aβ42 levels in genetically modified mice. Our findings are of concern in the context of AD and current discussions to re-examine BACE1 inhibitors as therapeutic and preventive agents. The data indicates that the BACE1/APP ratio primarily affects the balance between BACE1-mediated Aβ production and degradation and highlights a critical role to BACE1 in amyloid clearance that has previously been neglected.

## Materials and methods

### Plasmids and siRNAs

A human BACE1 construct (full length BACE1, isoform A; pcDNA3.1+/Zeo; Invitrogen), APP695 (with an N-terminal Myc-tag; pcDNA3.1+/Zeo; Invitrogen) and APP-C99 (with a C-terminal FLAG-tag; pcDNA3.1+/Zeo; Invitrogen) were used for transient overexpression in HEK293T cells. Point mutations were introduced by site-directed mutagenesis, using PfuUltra II Fusion HS (Stratagene/Agilent) followed by DpnI (NEB) digestion. All constructs were verified by DNA sequencing. For creating stably expressing SH-SY5Y cells, full-length human BACE1 (isoform A) and full-length human APP (isoform APP695, with an N-terminal Myc tag), in the mammalian expression vector pCEP4, Hygro (Invitrogen) were used. Mouse wild-type BACE1 construct with an N-terminal FLAG tag immediately following the propeptide cleavage was generated by overlap extension PCR and cloned in pSport6. LL/AA or D495R variants were then generated by PCR using reverse primers with the mutant sequence. Mock controls for corresponding plasmid backbones were used. For knockdown, siGENOME non-Targeting siRNA Pool #1 (D-001206-13-05), SMARTpool siGENOME Presenilin 1 (M-004998), and Presenilin 2 (M-006018) were used.

### Human brain samples

Post-mortem samples were collected from donors with a written informed consent for a brain autopsy and the material, and the use of the material for research purposes and the experimental protocols were approved by the Netherlands Brain Bank by the Implementing Letter regarding project nr. 836 "Diagnostic Potential of AP-clearance Intermediates in Elderly Subjects at Risk for Alzheimer's Disease", approved by the Director Netherlands Brain Bank, 26 Aug 2014, Royal Netherlands Academy of Arts and Sciences (KNAVV) acting for and on behalf of The Netherlands Brain Bank (NBB), a department of the Netherlands Institute for Neuroscience (NIN), whose registered office is at Meibergdreef 47, 1105 BA Amsterdam, The Netherlands.

Frozen samples from the temporal cortex from non-demented controls (n = 5) and confirmed Alzheimer disease Braak 4 to Braak 6 (n = 20) were prepared as previously described^65^. In brief, brain samples were thawed on ice, weighed and homogenized in buffer A (100 mM Tris–HCl, 150 mM NaCl, 2 × complete protease inhibitor cocktail (Roche)) using gentleMACS™ M Tubes/Dissociator at 4 °C (Miltenyi Biotech). TritonX-100, final concentration 1%, was added and samples were incubated for 1 h with agitation at 4 °C. Lysates were centrifuged at 10,621 rcf in a microfuge (Eppendorf) at 4 °C for 15 min to remove the nuclear fraction. Samples were measured with bicinchoninic acid assay (BCA assay, Thermo Fisher Scientific Inc., Pierce) and MSD assays.

### Mouse brain lysates

We complied with all relevant ethical regulations for animal tissue testing and research. Details on ethics approvals for animal studies are available from our co-authors of the laboratories in Germany that provided the material. Briefly, London APP Transgenic mice and wild-type littermates: All animal studies were conducted in compliance with European and German guidelines for the care and use of laboratory animals and were approved by the Central Animal Facility of the University of Mainz and the ethical committee on animal care and use of Rhineland–Palatinate, Germany.

BACE1 +/+, BACE +/− and BACE1 −/− mice: In agreement with the German animal welfare law all animal handling and care were performed according to the guidelines of the Christian-Albrechts-University of Kiel. The Ministry of Energy, Agri-culture, the Environment and Rural Areas Schleswig–Holstein approved animal experiments under the reference number V242–40,536/2016 (81–6/16).

Cortices of transgenic mice expressing London APP and their wild-type littermates were provided by Dr. Claus Pietrzik’s laboratory at the University of Mainz, Germany. Cortices of BACE1 +/+, BACE +/− and BACE1 −/− mice were provided by Dr. Paul Saftig’s laboratory in University of Kiel, Germany. All mice were on C57BL/6 strain genetic background and were 6-months of age when sacrificed. Frozen mouse brains were thawed on ice, weighed, and homogenized in the homogenization buffer (100 mM Tris–HCl pH: 7.5, 150 mM NaCl and 2 × complete protease inhibitor cocktail (Roche)) using Dounce homogenizer. 10% Triton-X was added to the homogenates (final concentration: 1%). Brain homogenates were lysed at 4 °C for 1 h on a rotator. Lysates were centrifuged at 10,621 rcf in a microfuge (Eppendorf) at 4 °C for an hour to remove the debris. Supernatants were collected and diluted in the appropriate buffers for BCA, Western blot and MSD assays.

### Cell culture and transfection

Human Embryonic Kidney (HEK293T) cells (DSMZ No. ACC305; DSMZ, Braunschweig, Germany) cells were grown in DMEM (High glucose (4.5 g/l), 10% fetal bovine serum (FBS), 2 mM glutamine, 1 mM pyruvate) in a humidified incubator at 37 °C 5% CO_2_. For transient transfection experiments, cells were seeded on 6-well plates (Fisher) coated with poly-D-lysine (Sigma) and transiently transfected (with the plasmids indicated for each experiment) 20–24 h later by using TransFectin according to the protocol provided by the manufacturer (Biorad). 24 h after transfection, media of the cells were changed, and cells were conditioned for 16 h before sample collection.

Human neuroblastoma (SH-SY5Y) cells (DSMZ No. ACC209; DSMZ, Braunschweig, Germany) stably overexpressing BACE1, APP or APP-C99 were cultured in DMEM/F12 (10% fetal bovine serum (FBS), 2 mM L-glutamine, 1 mM sodium pyruvate) in a humidified incubator at 37 °C 5% CO_2_. Stable cell lines were selected with 250 µg/ml Hygromycin B (Milipore). For BACE1 localization, cells were seeded on 6-well plates (Fisher) and transfected with FuGENE HD (Promega) after 24 h. 72 h after the transfection, cells were harvested. For PS1 and/or PS2 knockdown experiments, cells were seeded on 6-well plates (Fisher) and treated with either control, PS1 or PS2 siRNA (concentration of the siRNA depended on the experiment) 24 h later by using RNAiMax according to the protocol provided by the manufacturer (Invitrogen). 72 h after the treatment, cells were harvested. For ICC experiments, cells were seeded on 24-well plates (Fisher) and the same protocols were applied.

### Sample preparation

For all experiments performed, cells were harvested on ice. Conditioned media were centrifuged at 2000 rpm at 4 °C for 10 min and Aβ34, Aβ40 and Aβ42 levels were quantified by ELISA. Cells were washed with cold PBS and lysed with Whole Cell Extract Buffer (25 mM HEPES (pH 7.7), 0.3 M NaCl, 1.5 mM MgCl_2_, 0.2 mM ethylenediaminetetraacetic acid, 0.1% Triton-X-100, 0.5 mM dithiothreitol, 4 mM NaF, 0.1 mM Na_3_VO_4_, 1 mM PMSF, Complete Protease Inhibitor Cocktail (Roche)) at 4 °C for 60 min. Cell lysates were cleared from nuclear material by centrifugation at 10,000 rpm at 4 °C for 15 min and protein levels were detected by Western Blot.

### Western blot analysis

Samples were prepared by adding LDS loading buffer and 2-Mercaptoethanol to the cell lysates according to the protocol provided by the manufacturer (Invitrogen). The proteins were solubilized and denatured by heating the samples to 70 °C for 10 min. Proteins were separated on 4–12% Bis–Tris gradient gels (Invitrogen) and were transferred to 0.45 µm nitrocellulose (Biorad) or polyvinylidene difluoride (PVDF) (Millipore) membranes at 400 mA at 4 °C for 2.5 h. Proteins were detected by the antibodies indicated in the antibodies section. The primary and secondary antibodies were used in phosphate-buffered saline. Signals were recorded on ImageQuant LAS 500 and LAS 600 (GE Healthcare Life Sciences).

The primary antibodies used for Western Blot analysis were the following: anti-BACE1 1:2,000 dilution (monoclonal D10E5, Cell Signaling), anti-BACE1 1:2,000 dilution (B0681, Sigma-Aldrich), anti-actin 1:5,000 dilution (monoclonal mab1501, Millipore), anti-sAPPβ 1:2,000 dilution (IBL), and anti-APP ectodomain 22C11 1:10,000 dilution (Millipore), anti-flag 1:1,000 dilution (M2, F1804, Sigma-Aldrich), anti-PS2 (ab51249, Abcam), and anti-PS1 1:10,000 dilution (ab76083, Abcam).

The secondary antibodies used for Western Blot analysis were the following: anti-mouse- and anti-rabbit-horseradish peroxidase 1:10,000 dilution (Promega).

Quantification of the Western Blots were performed with ImageJ and all protein levels were normalized to actin.

All gels and blots used in figures are in compliance with the digital image and integrity policies (https://www.nature.com/srep/journal-policies/editorial-policies#digital-image).

Where cropped gels/blots are displayed, respective full-length gels and blots are included in a Supplementary Information file.

### Meso scale discovery (MSD) assay

Custom-printed 4-plex plates were used as described previously^20^. Plates were blocked with 150 µl 5% MSD Blocker A solution for an hour at room temperature with gentle shaking and washed 3 times with 250 µl PBS-T (0.05% tween). Peptide calibrators were diluted in MSD Diluent 35. Plates were loaded with samples and calibrators together with SULFO-TAG™ 4G8 or 6E10 detection antibody diluted in MSD Diluent 100 and incubated overnight at 4 °C with gentle shaking. After three washes with 250 µl PBS-T, 150 µl 2 × MSD read buffer was added to the wells. Plates were read by an MSD QuickPlex SQ 120 Imager and data were analyzed by MSD Workbench® software.

### Sandwich-based Enzyme-Linked Immunosorbent Assay (ELISA)

5 μg/ml monoclonal anti-Aβ34 (226), anti-Aβ40 (G2-10) or anti-Aβ42 (G2-13) capture antibody in 100 mM sodium carbonate (pH 9.6) were used to coat the 96-well Nunc™ plates (Thermo Fisher Scientific Inc.). The sealed plates were incubated overnight at 4 °C with gentle shaking. Plates were washed 5 times 10 min with PBS-T washing buffer (1.1 mM NaH_2_PO_4_, 8.5 mM Na_2_HPO_4_, 13.7 mM NaCl, (pH 7.4), 0.1% Tween-20). 250 μl Stabil Coat®Immunoassay Stabilizer (SurModics Inc.) was used for blocking and plates were incubated for 2 h at room temperature with gentle shaking. 50 μl of 0.075 μg/ml detection antibody, W02-biotin, in assay buffer (90% 11 mM NaH_2_PO_4_, 85 mM Na_2_HPO_4_, 137 mM NaCl, (pH 7.4), 0.5% Tween-20, 1.5% BSA, 0.01% Thimerosal, and 10% SeaBlock blocking buffer (Thermo Fisher Scientific Inc.) was loaded to the wells together with 50 μl sample (cell media) or calibrator (synthetic peptide standards diluted in DMEM or DMEM/F12). After overnight incubation at 4 °C with gentle shaking, plates were washed 5 times for 10 min with PBS-T washing buffer. For Aβ40 ELISA, 100 μl Mono-HRP-conjugated-streptavidin (Pierce) (0.1 μg/ml) in Mono-HRP buffer (11 mM NaH_2_PO_4_, 85 mM Na_2_HPO_4_, 137 mM NaCl, (pH 7.4), 0.05% Tween-20, 6% PEG) or for Aβ34 and Aβ42 ELISA (for higher sensitivity), 100 μl Poly-HRP-conjugated-streptavidin (Pierce) (1:20,000 dilution) in Poly-HRP buffer (1.1 mM NaH_2_PO_4_, 8.5 mM Na_2_HPO_4_, 13.7 mM NaCl, (pH 7.4), 0.1% Tween-20, 5% BSA)) was added to the wells. Plates were incubated for 1 h at room temperature with gentle shaking and washed 5 times for 10 min with PBS-T washing buffer. For the initiation of enzymatic reaction, 100 μl 1-Step™ Ultra TMB-ELISA Substrate (Thermo Fisher Scientific Inc.) solution was added to the wells and the plates were incubated at room temperature in the dark for up to 30 min. To stop the reaction, 50 μl 1 M H_2_SO_4_, per well, was added. Using Synergy H1, BioTek Instruments Inc. plate reader, absorbance at 450 nm and 630 nm as a reference was measured. The data analysis was performed with Gen5 BioTek®software. For the fitting of standard curves obtained from the absorbance of calibrators, a non-linear four-parameter logistic fit without weighting was used as follows

$$y={b}_{2}+\frac{{b}_{1}-{b}_{2}}{1+{(\frac{x}{{b}_{3}})}^{{b}_{4}}}$$

where y is signal, x is concentration, b_2_ is estimated response at the infinite concentration, b_1_ is estimated response at zero concentration, b_3_ is mid-range concentration and b_4_ is slope factor.

### Immunocytochemistry

For all immunofluorescence experiments, 12 mm coverslips were used (Fisherbrand™ Catalog# 12CIR1602811G). SH-SY5Y cell lines were fixed with 4% formaldehyde in phosphate buffered saline. Cells were then permeabilized with 1% Triton X-100 for 10 min and blocked immediately for 30 min with 2% bovine serum albumin (BSA) in phosphate buffered saline solution. After blocking, coverslips were incubated with the primary antibody overnight at 4 °C. The following day, the primary antibody was washed off, the coverslips were washed 3 times in 2% BSA buffer and were then incubated with secondary antibody for 30 min. After incubation, coverslips were washed in PBS and nuclei were stained with NucBlue (ThermoFisher catalog #R37606). In order to visualize the actin network some cells were stained with Phalloidin for 20 min according to the manufacturer’s instructions (ThermoFisher catalog #A22287). Coverslips were then mounted onto microscope slides using the Aqua-Poly/Mount media (Polysciences Catalog #18,606–20).

The primary antibodies used for immunofluorescence were the following: anti-PS1 antibody dilution 1:100 (Invitrogen, MA1-752), anti-PS2 antibody 1:50 dilution (Abcam ab15549), anti-EEA1 antibody 1:200 dilution (Cell Signaling #3288), anti-LAMP1 antibody 1:200 dilution (Cell Signaling #9091), and anti-BACE1 C-term antibody 1:100 dilution (Millipore MAB5308).

The secondary antibodies were acquired from Life Technologies: goat anti-rabbit IgG cross adsorbed Alexa Fluor 647 (ThermoFisher catalog #A-21245; diluted 1:10,000), goat anti-mouse IgG cross absorbed Alexa Fluor 568 (ThermoFisher catalog #A-11031) or goat anti-mouse IgG cross absorbed Alexa Fluor 488 (ThermoFisher catalog #A-11001).

### Confocal microscopy and image analysis

Single- or double-immunolabeled (Alexa Fluor-488, -568 or -647) samples were analyzed at the Imaging & Molecular Biology Platform (IMBP; McGill Life Sciences Complex) using a TCS SP8 multi-photon confocal microscope (Leica) with 63x/1.40 oil-immersion objectives (Leica, Wetzlar, Germany). Samples were excited with Coherent Chameleon Vision II multiphoton at 730 nm (2660 mW) for DAPI imaging. For each sample, 12–30 z-stack images were acquired using the same laser intensity settings for quantification. Z-stack images were processed using Image-J (Rasband, W.S., ImageJ, U.S. National Institutes of Health, Bethesda, Maryland, USA, https://imagej.nih.gov/ij/, 1997–2018) and total cell fluorescence was quantified with the analyze tool. To better visualize BACE1 localization, a heatmap was generated using Fire LUT in Image-J. The IMARIS Image Analysis Software (Bitplane (Oxford Instruments), MA, USA) software was used for cross-sectional analysis. BACE1 colocalization with EEA1 and LAMP1 were analyzed using ImageJ plugin JACoP^66^.

### Matrix-assisted laser absorption ionization mass spectrometry (MALDI-MS)

Samples were first immunoprecipitated (IP). For each IP (4 °C, 18 h), 0.5 mL of conditioned cell culture supernatant was combined with 5 µg W02 (anti-Ab antibody) and 25 µL protein-G sepharose beads (GE Healthcare) in PBS (1 mL final volume). The samples were sequentially washed with PBS, followed by 10 mM Tris pH 7.5; 150 mM NaCl; 0.2% NP-40; 2 mM EDTA, followed by 10 mM Tris pH 7.5; 500 mM NaCl; 0.2% NP-40; 2 mM EDTA, followed by three-times PBS and finally three-times 100 mM ammonium acetate (pH 7.4). The IPs were eluted twice using 350 µl volumes of 50% acetic acid. The vacuum-dried samples were resuspended in 10 µl of TA30 (33% acetonitrile and ultrasonicated. Samples were mixed 1:1 with α-cyanocinnamic acid matrix (CCA, Bruker Daltonics; 20 mg/mL in TA30) and applied to ground steel MALDI targets using the dried droplet method. Mass spectra were recorded on an UltrafleXtreme MALDI-TOF/TOF system (Bruker Daltonics) using the reflector positive 900–4500 method (ion source 1 = 25 kV; ion source 2 = 22.30 kV; lens = 9.40 kV; reflector = 26.45 kV; reflector 2 = 13.40 kV; pulsed ion extraction = 150 ns) and flexControl v1.4 and flexAnalysis v1.4 software. Ion intensity was evaluated by averaging four measurements of 500 shots each (i.e., 2000 shots total per sample).

### Statistical analysis

For all experiments, different conditions were analyzed by one factor ANOVA (between subject design) or two factor ANOVA. Pairwise comparisons were performed either with Dunnet’s or Tukey’s post-hoc tests. The statistical analysis was run by GraphPad Prism 5. For human brain samples, Welch’s t-tests were performed. Data that was not normally distributed (Gaussian normality test) were analyzed with non-parametric tests. Spearman’s correlation was performed to test the correlation between BACE1 and Aβ34 levels.

### Ethics approval and consent to participate

Prior to starting the study, ethical approvals have been obtained. The study was conducted in accordance with Helsinki Declaration as revised in 2013 and performed in accordance with respective guidelines. The experimental protocols were approved by The Netherlands Brain Bank (NBB) where the brain post-mortem samples were obtained from, i.e. The Netherlands Institute for Neuroscience, Amsterdam (open access: www.brainbank.nl). All material has been collected from donors having provided written informed consent for a brain autopsy and the use of the material and for whom. Clinical information for research purposes had been obtained by the NBB.

## Supplementary Information

Supplementary Information 1.

Supplementary Information 2.

## Data Availability

All experimental protocols were approved by the respective named institutional and/or licensing committee. All methods were carried out in accordance with relevant guidelines and regulations. All methods are reported in accordance with ARRIVE guidelines (https://arriveguidelines.org). Data and material from this study will be made available upon reasonable request to the corresponding author (gerhard.multhaup@mcgill.ca).
