# Supplementary material for: Mechanisms of amyloid-β34 generation indicate a pivotal role for BACE1 in amyloid homeostasis
Source: Sci Rep. 2023 Feb 7;13:2216. doi: 10.1038/s41598-023-28846-z (PMC9905473; doi:10.1038/s41598-023-28846-z)

**Mechanisms of amyloid-β34 generation indicate a pivotal role for BACE1 in amyloid homeostasis**

**Supplementary Information – Uncropped gels**

**Figure 2a. Aβ34 levels in AD post-mortem brain and in mouse brain tissue correlated with altered BACE1 expression and enhanced Aβ40 and Aβ42 levels**

APP APP – actin


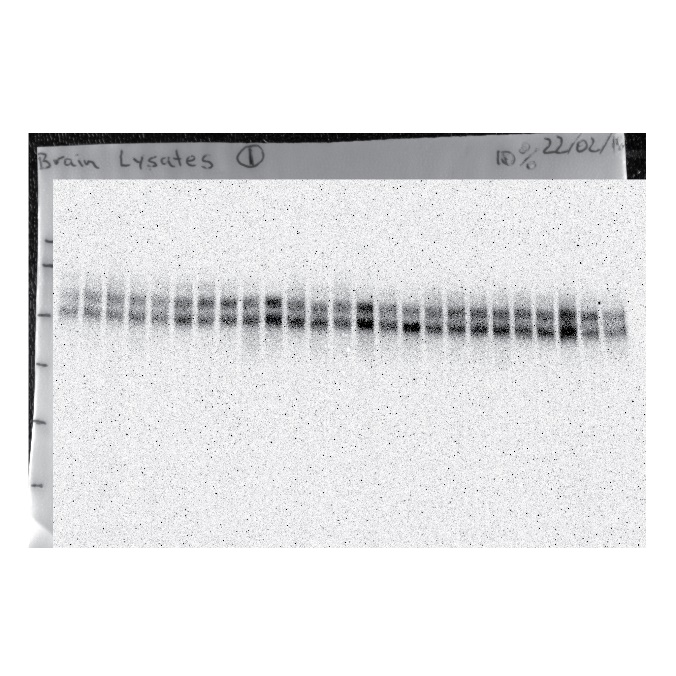

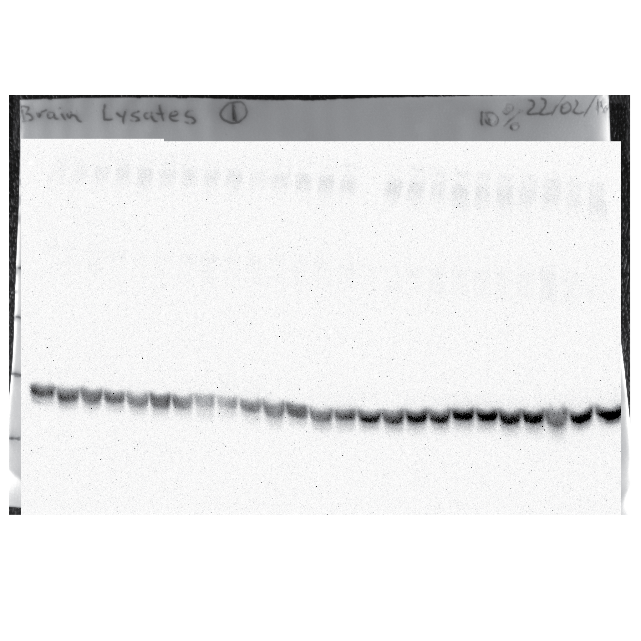


BACE1 BACE1 – actin


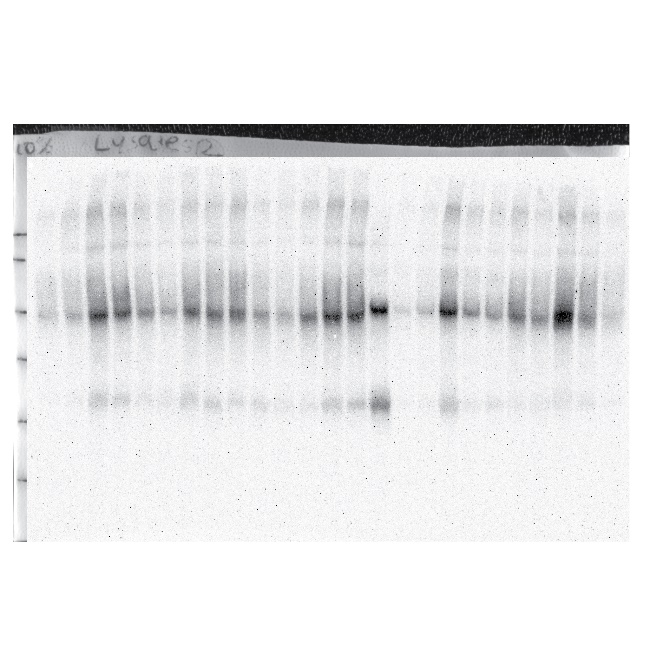

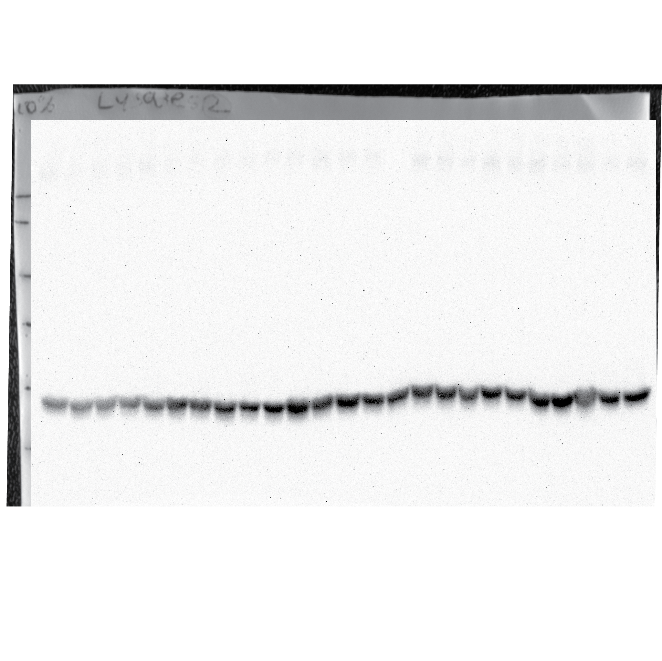


**Figure 3a. BACE1 overexpression and co-expression with APP-C99 enhanced Aβ34 production** **from** **Aβ40 and Aβ42**

APP BACE1


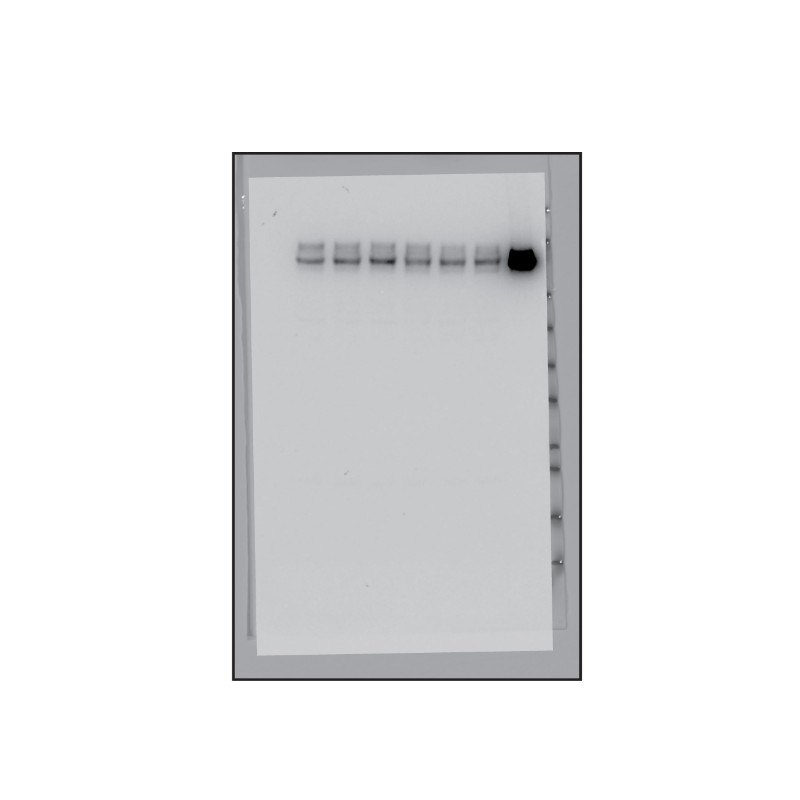

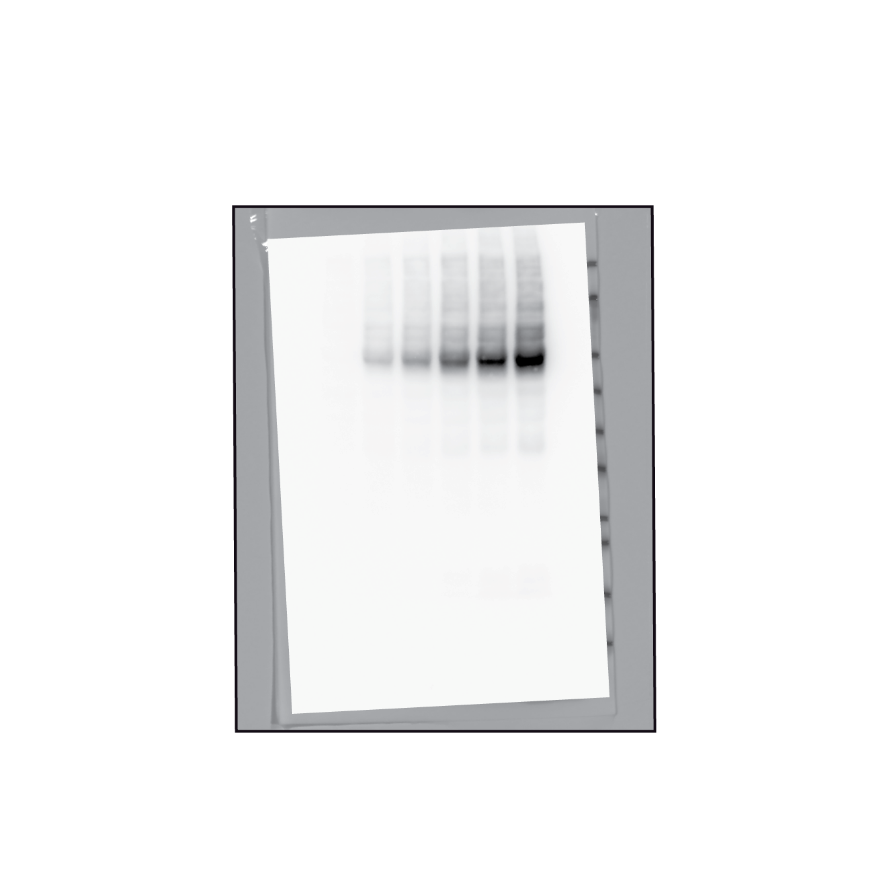


sAPPbeta sAPPtotal actin


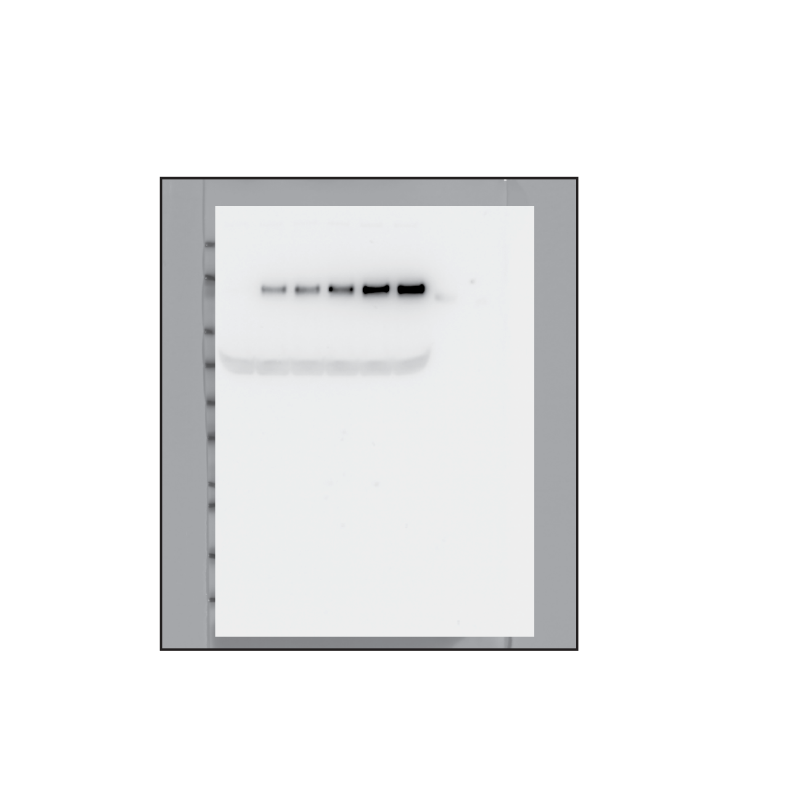

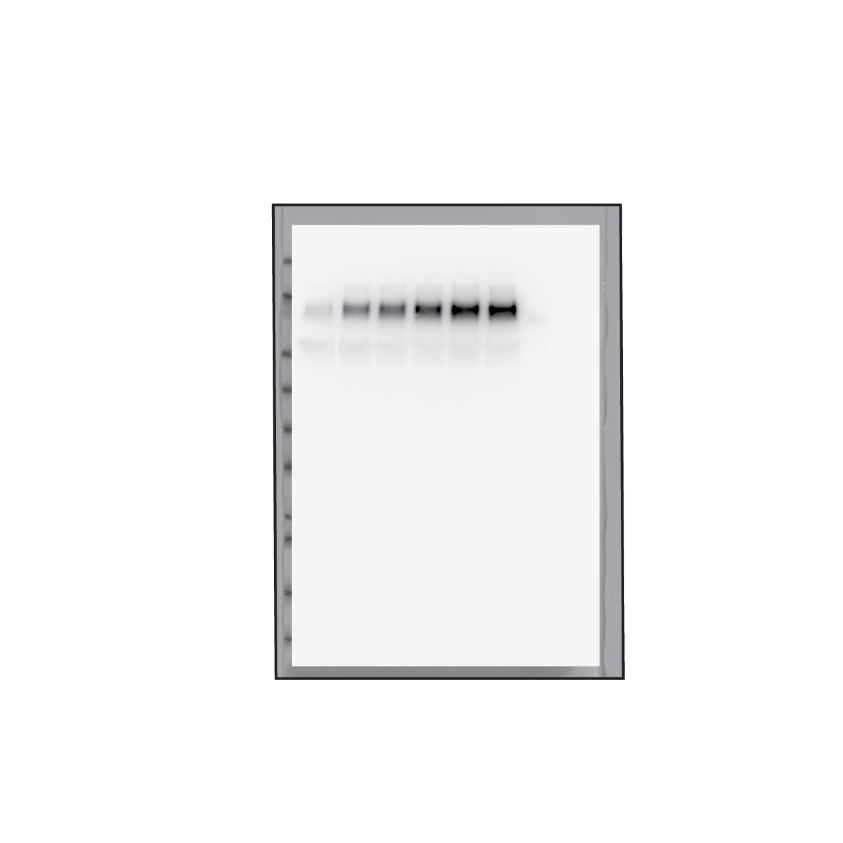

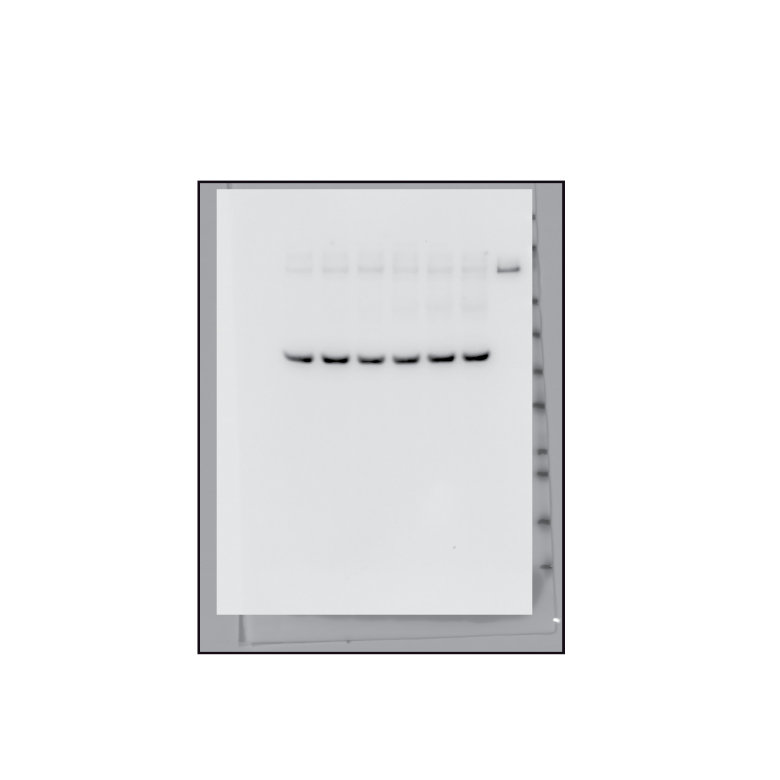


**Figure 3b**

APP BACE1


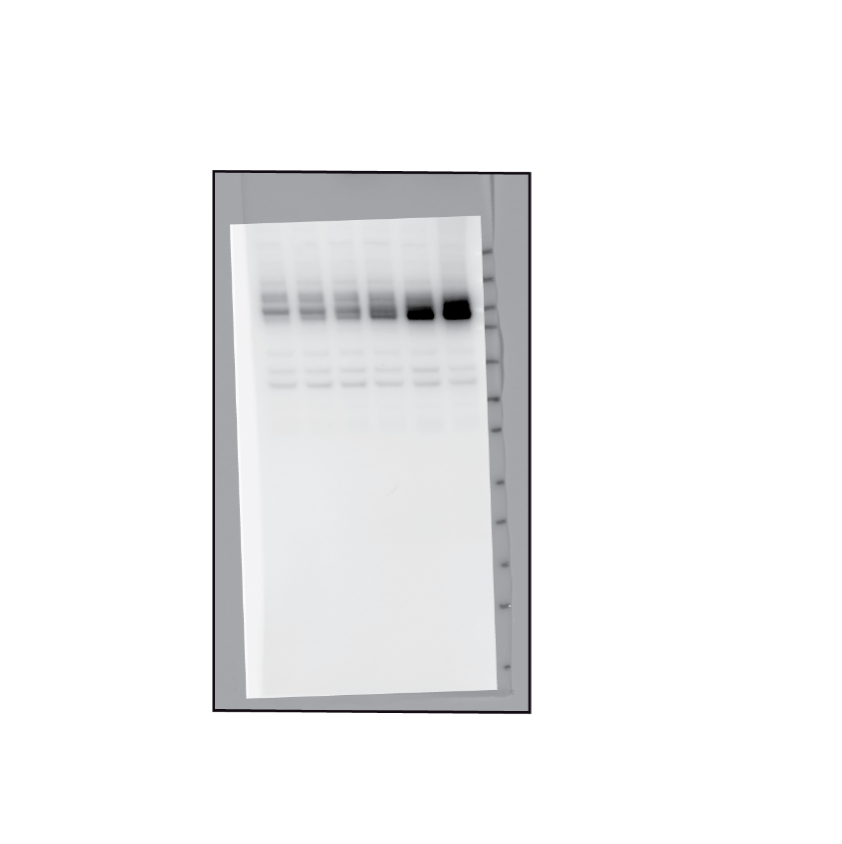

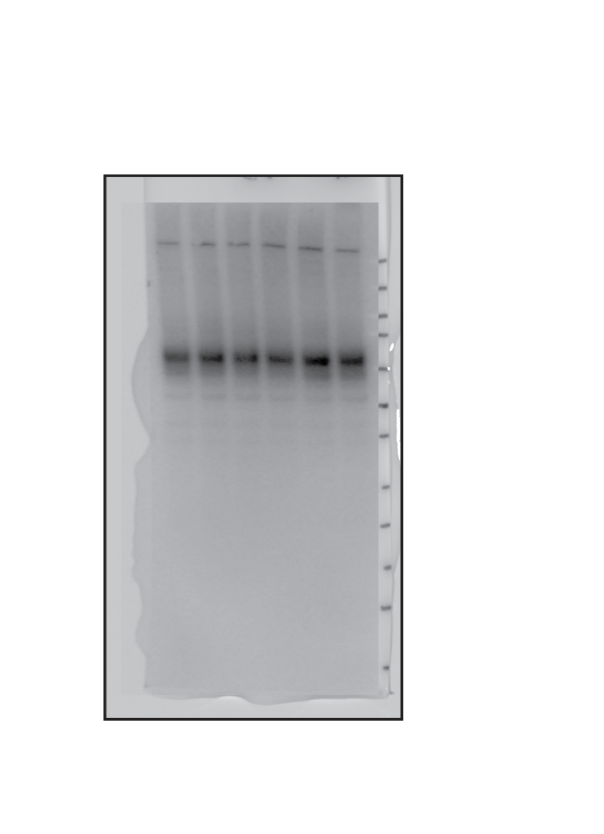


sAPPbeta sAPPtotal actin


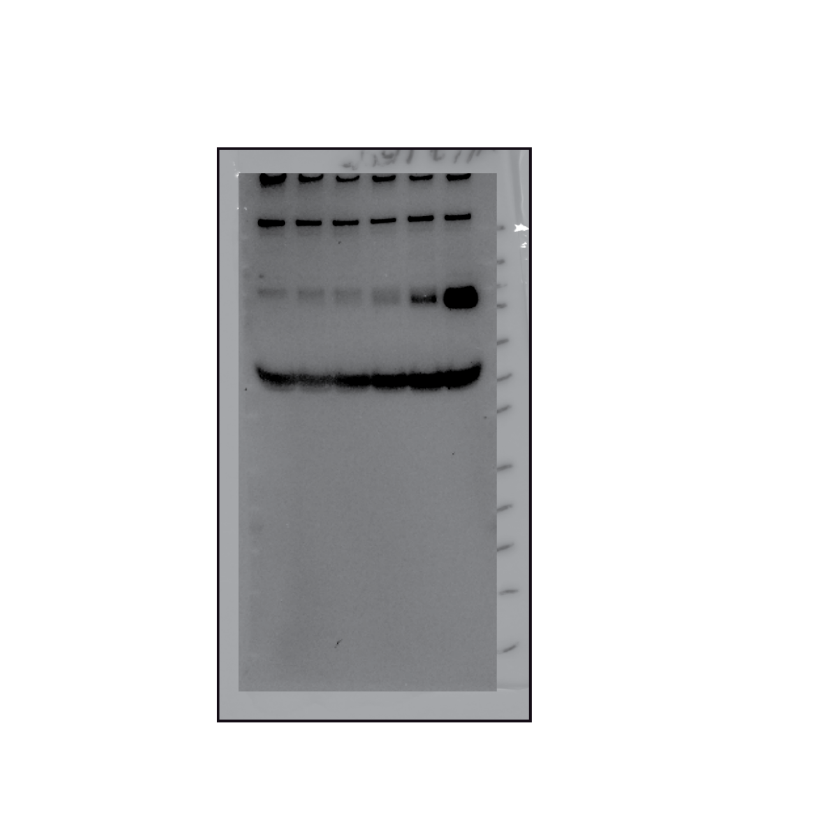

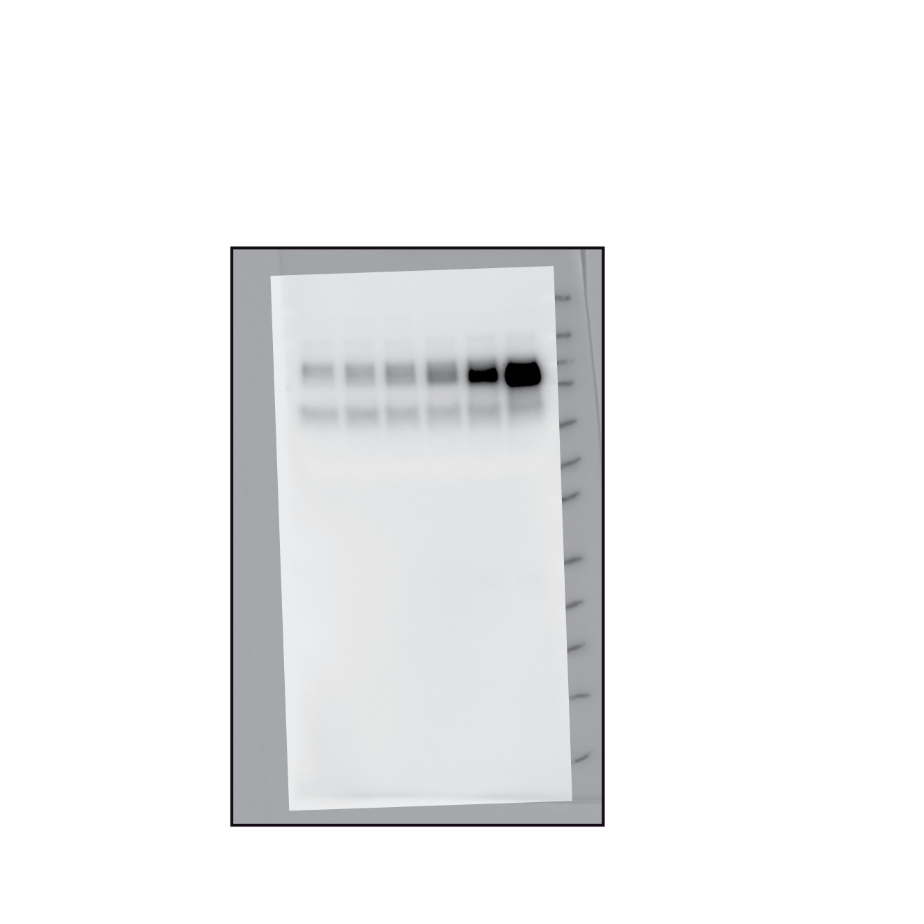

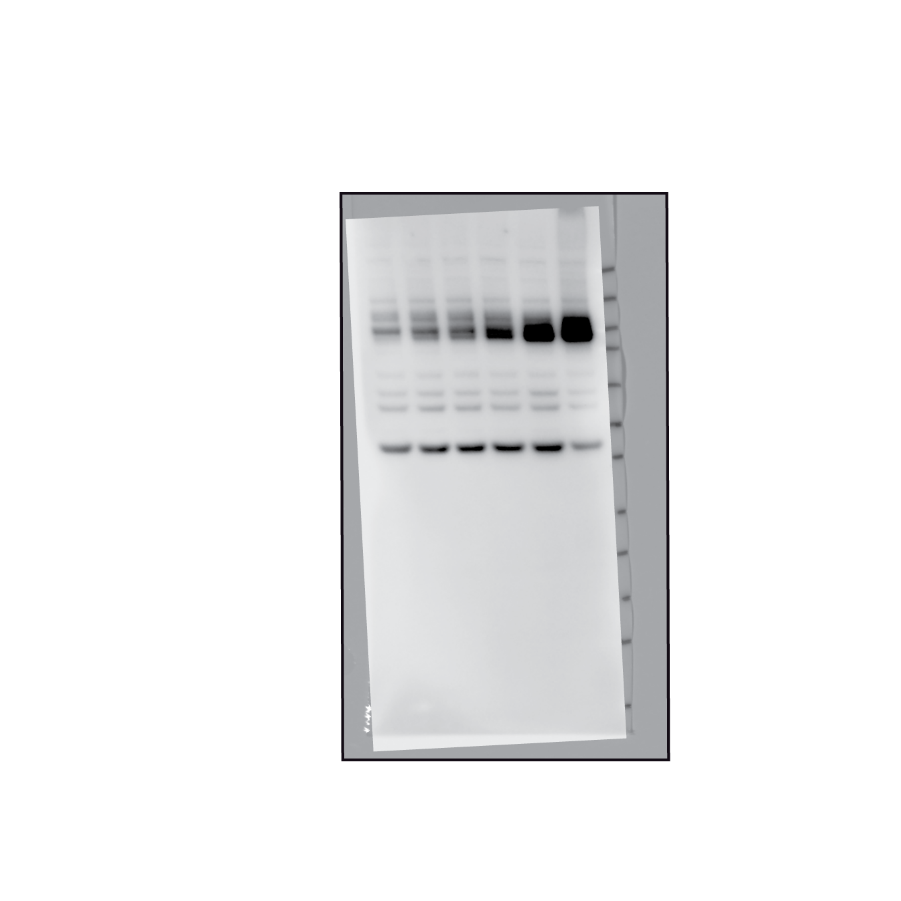


**Figure 3d**

BACE1 Flag – APP-C99


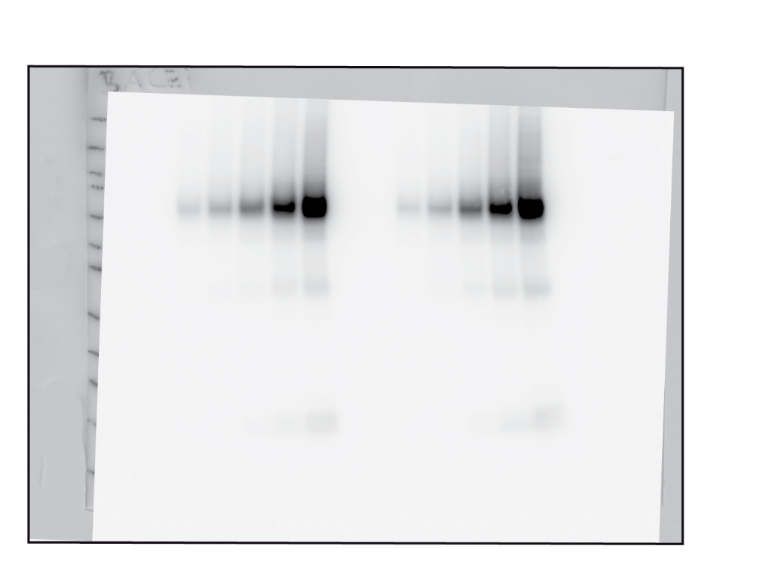

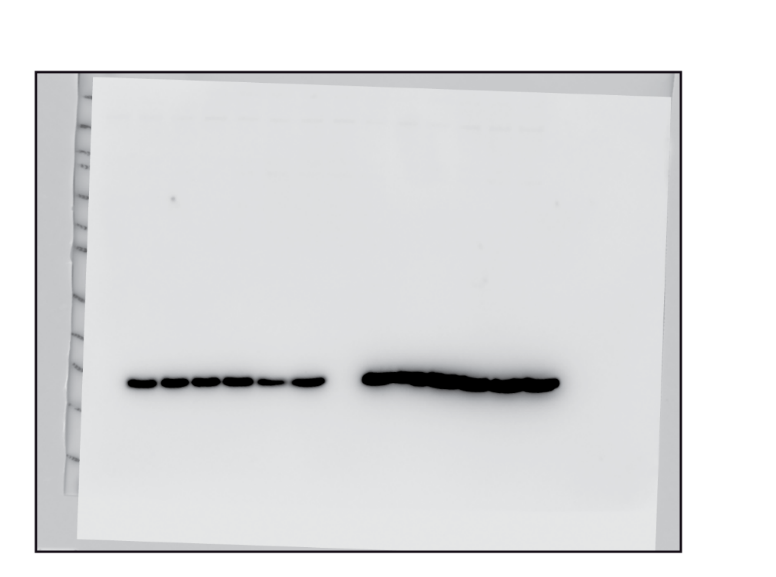


Actin


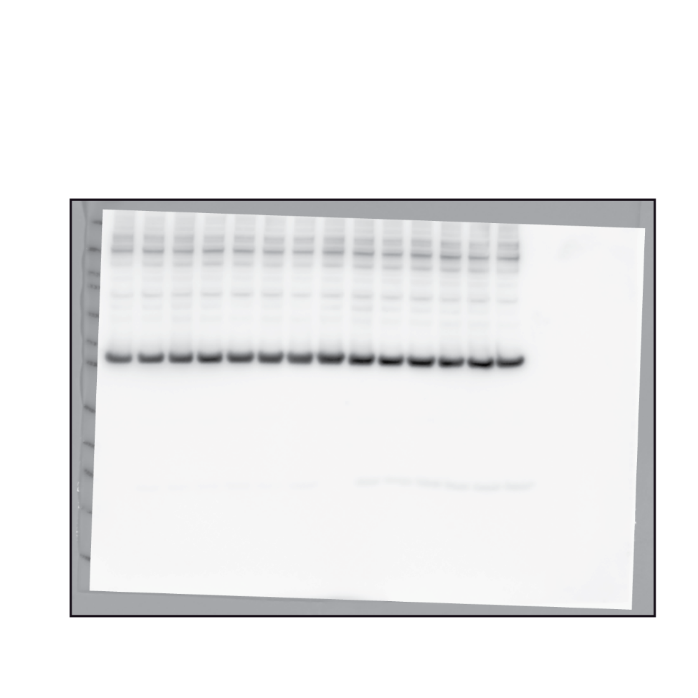


**Figure 3e**

BACE1 Flag – APP-C99


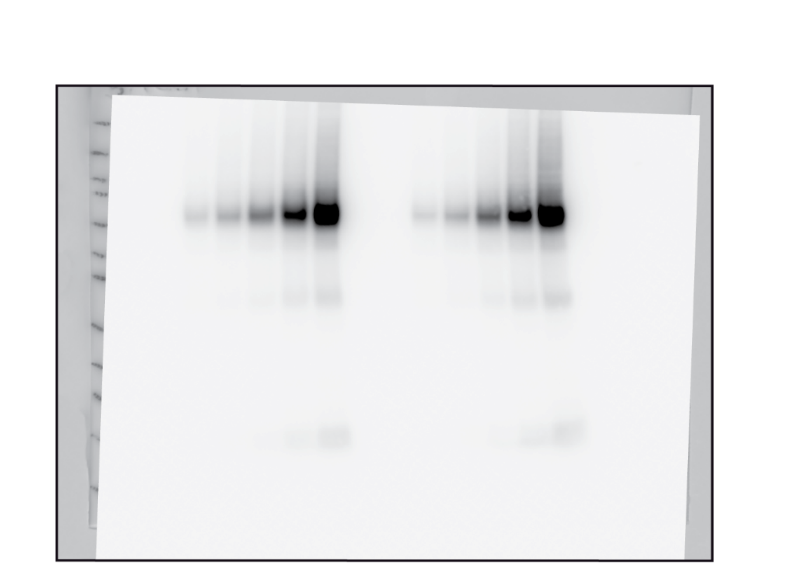

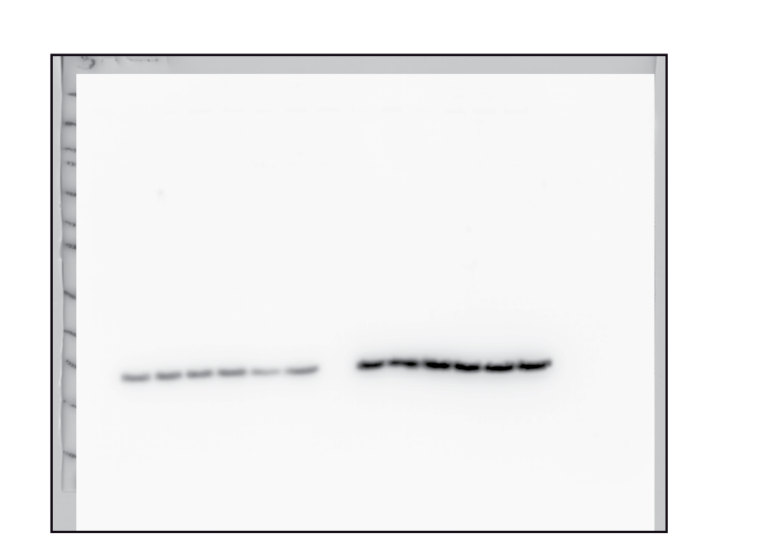


Actin


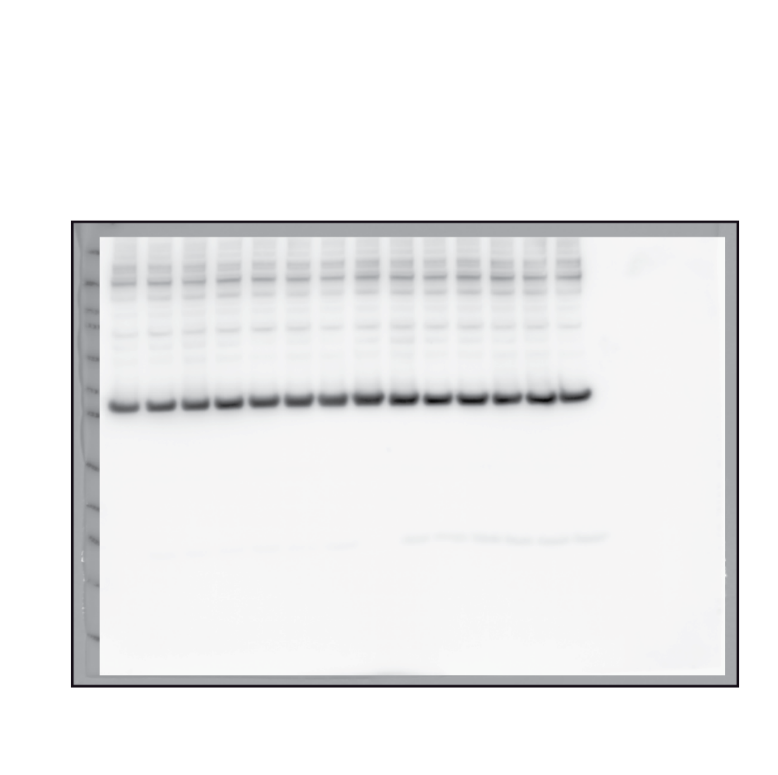


**Figure 4a. Altered localization of BACE1 to the endo-lysosomal system affected Aβ34 production**

BACE1 APP


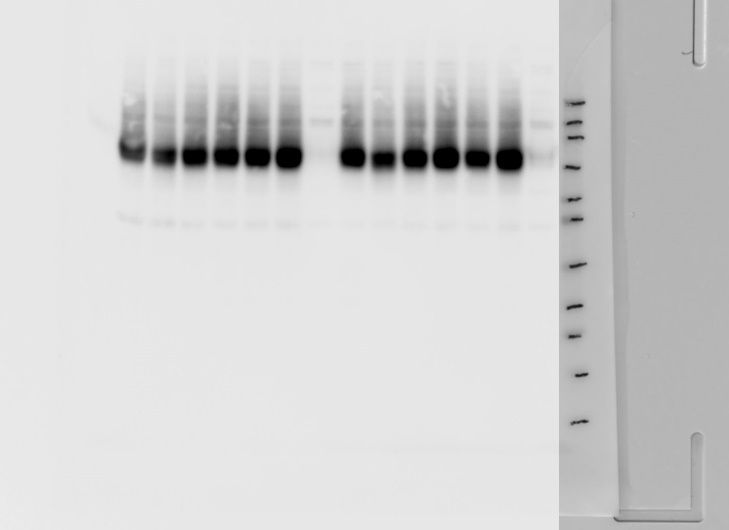

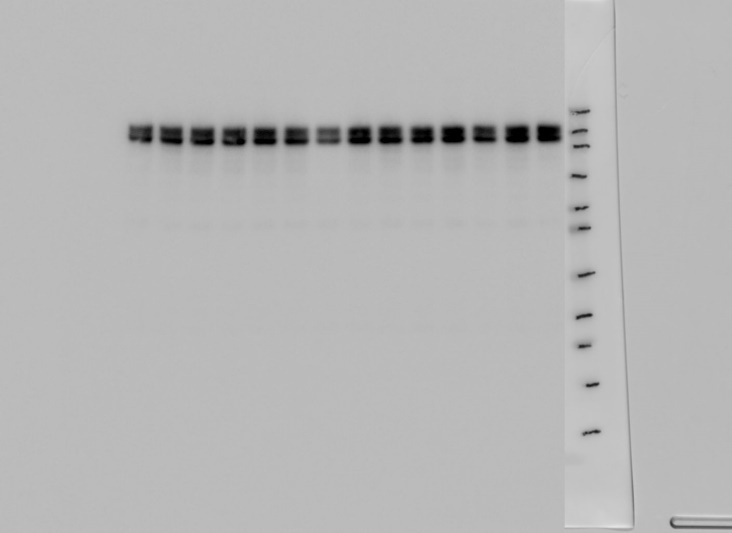


Actin


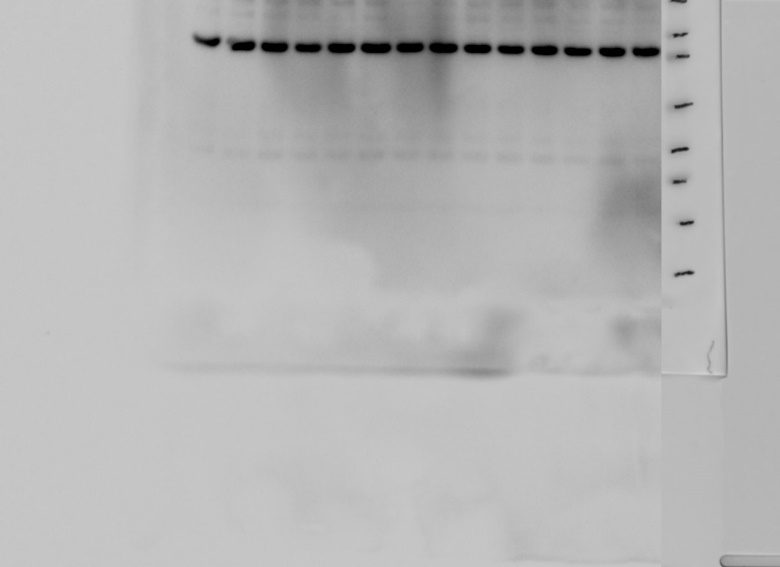


**Figure 4f**

BACE1 APP


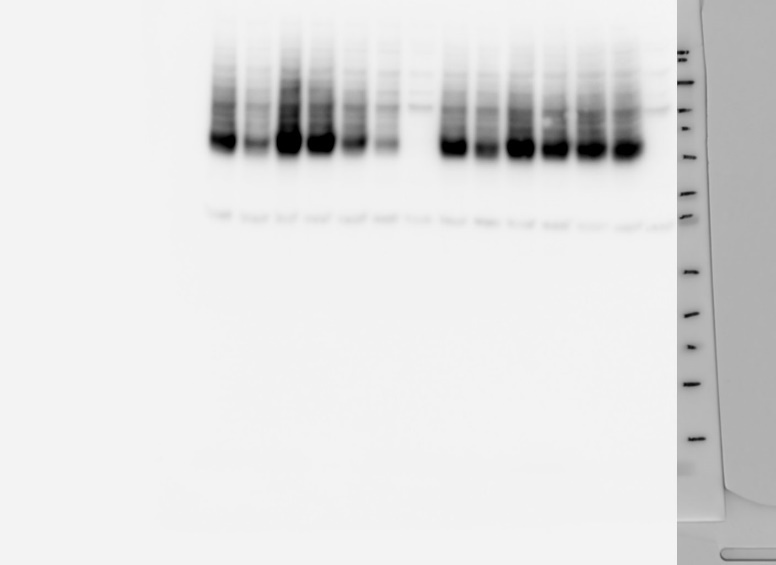

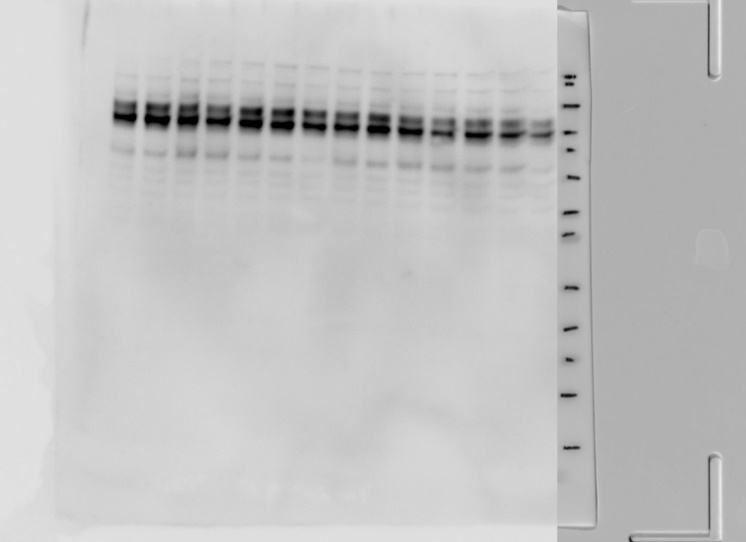


Actin


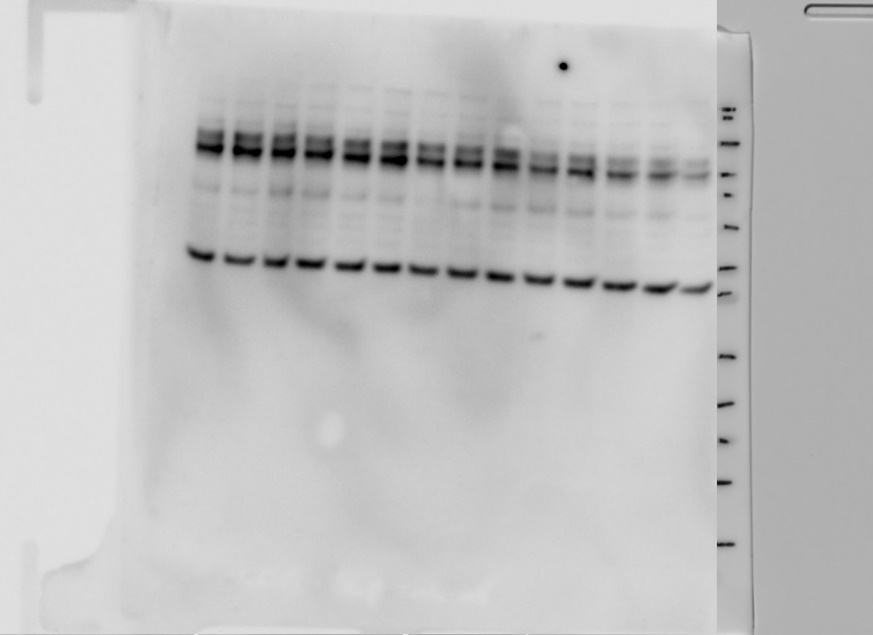


**Figure 5a.** **PS2 but not PS1 knockdown reduced Aβ34 levels from BACE1 overexpressing cells**

BACE1


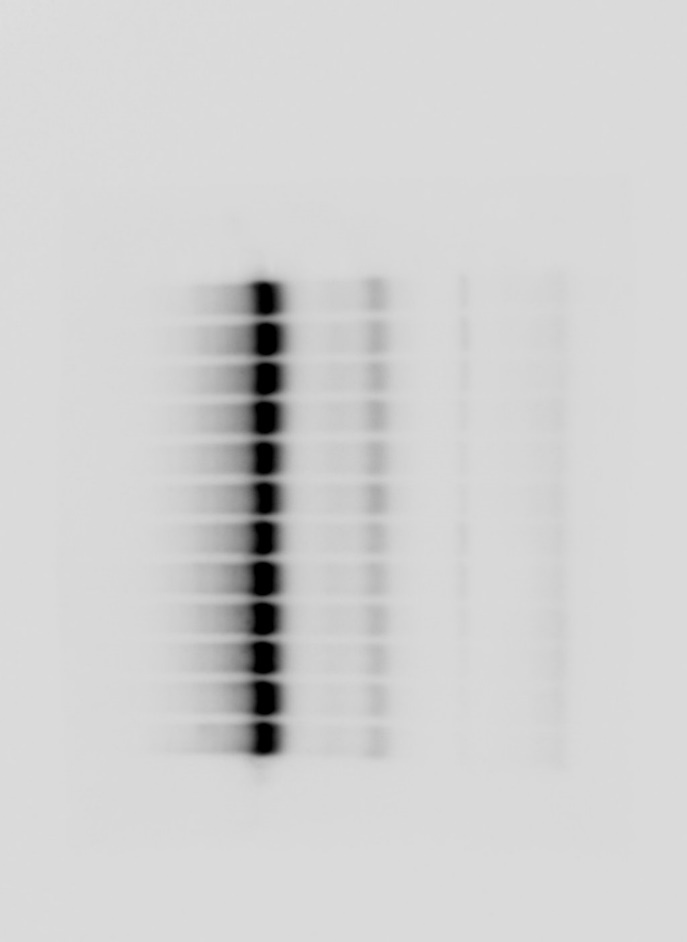


PS2 PS2 – actin


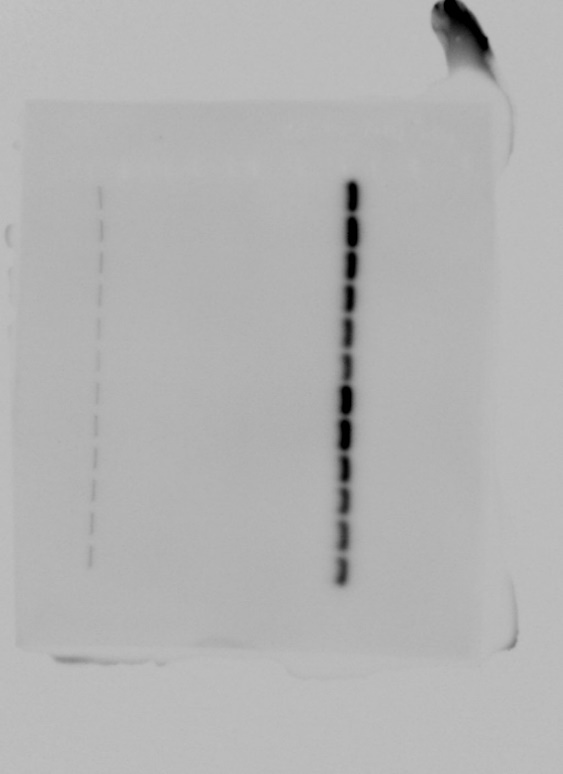

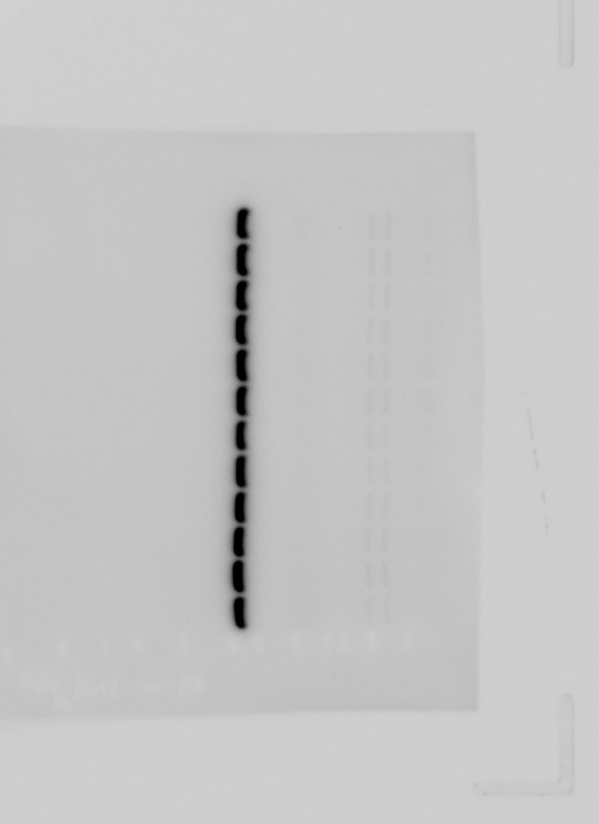


PS1 PS1 – actin


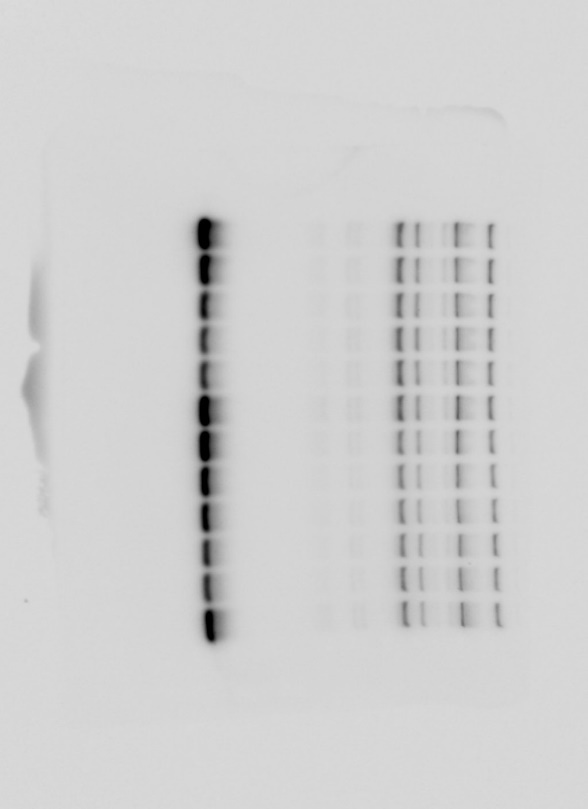

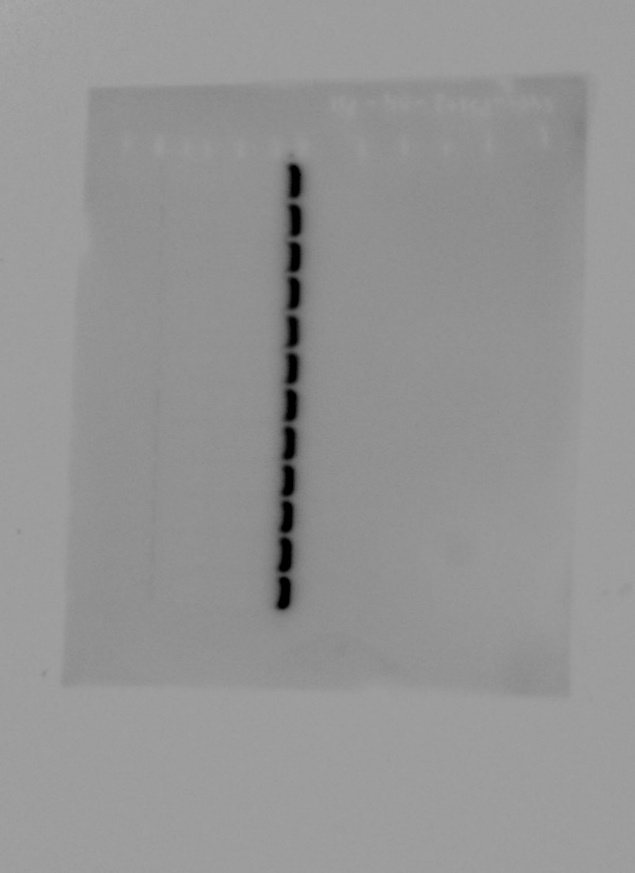


**Supplementary Figure 1a. Relative BACE1 levels in wild type and London APP Transgenic mice**

APP BACE1


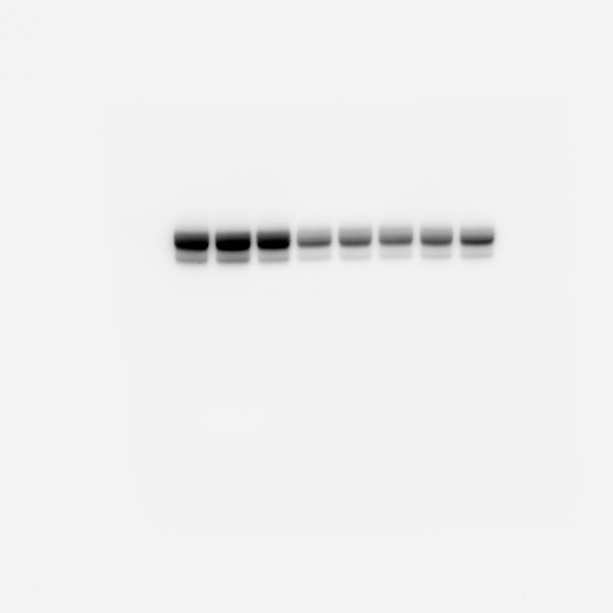

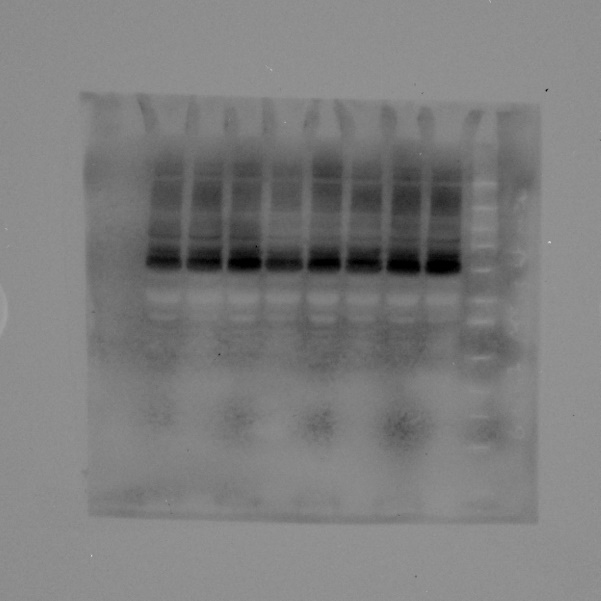


actin


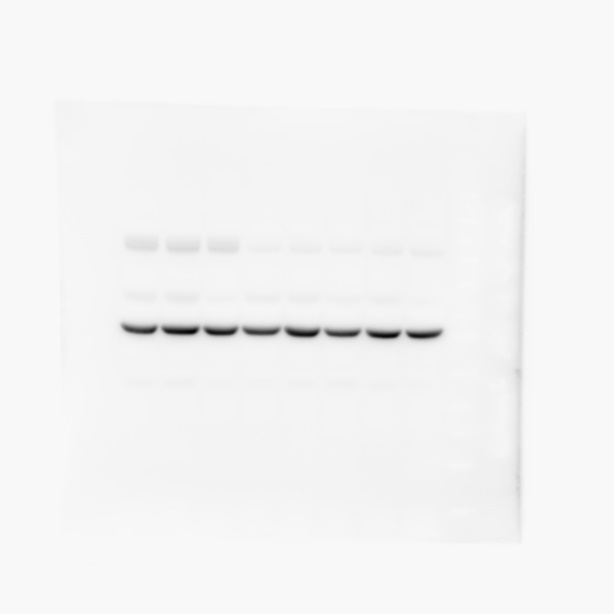


**Supplementary Figure 1b**

APP BACE1


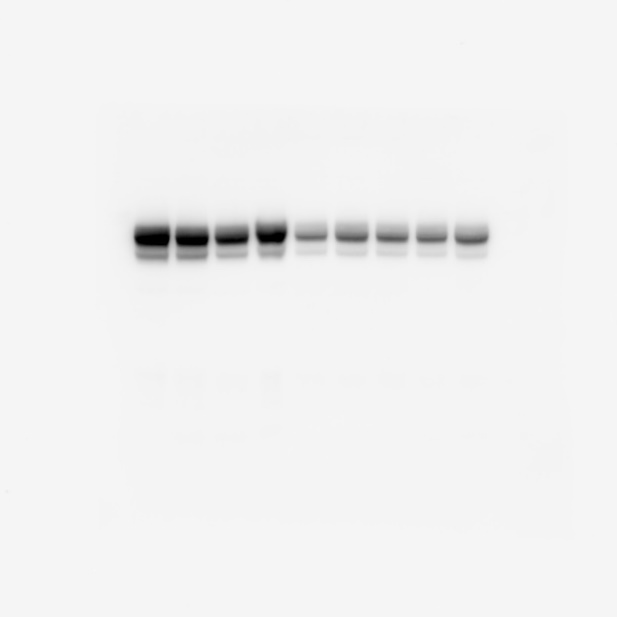

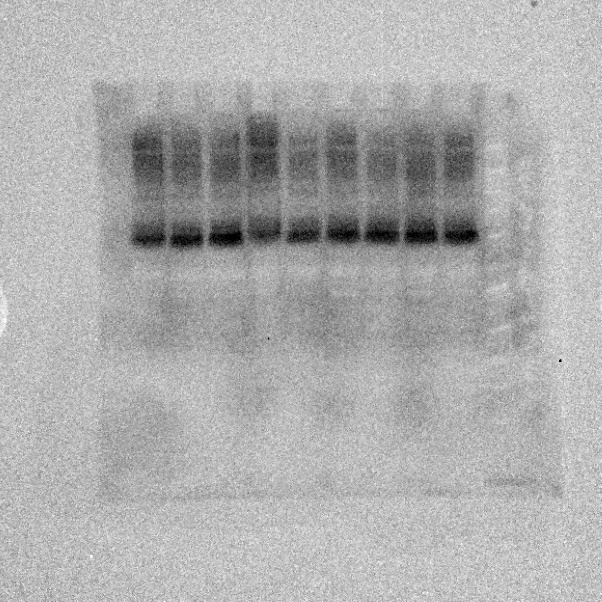


actin


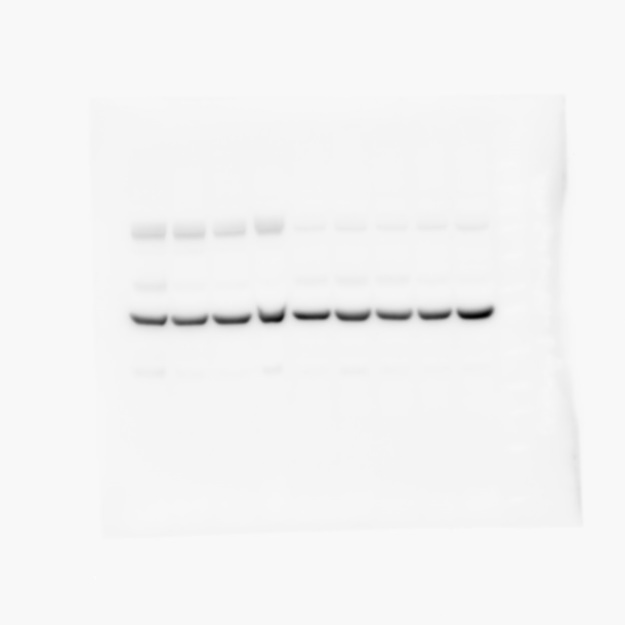


**Supplementary Figure 6a and 6e. Unlike PS1, PS2 knockdown leads to reduced Aβ34 levels in BACE1 overexpressing cells**

BACE1


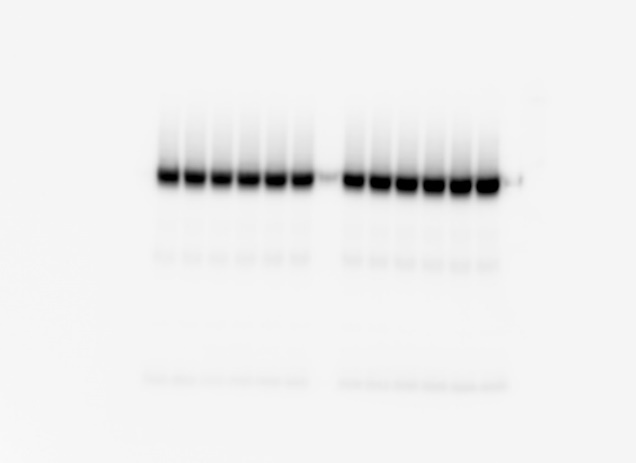


PS1 PS1 – actin


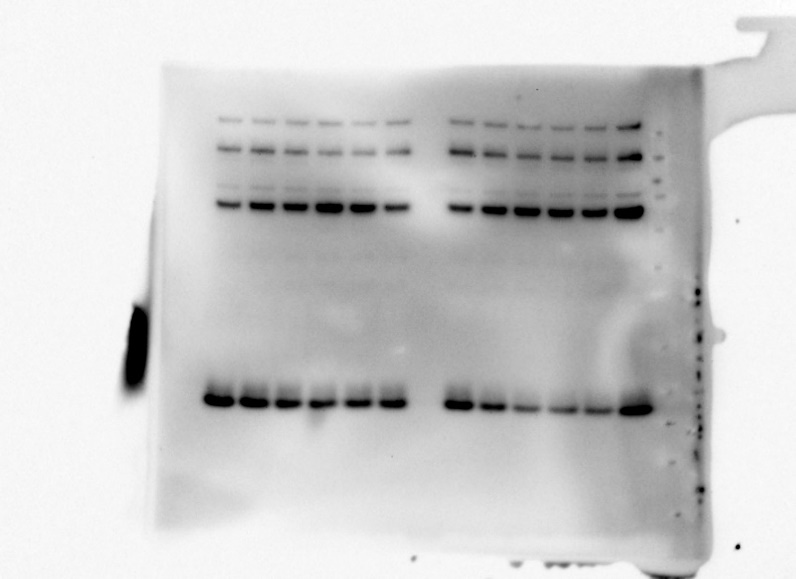

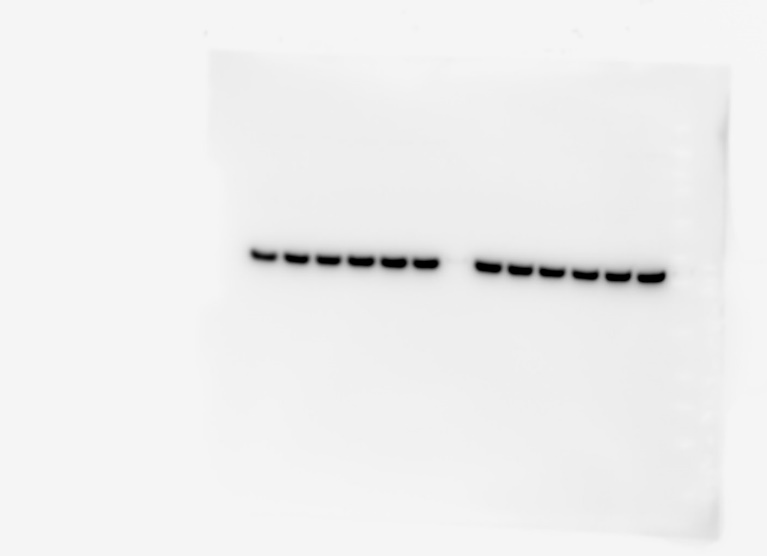


PS2 PS2 – actin


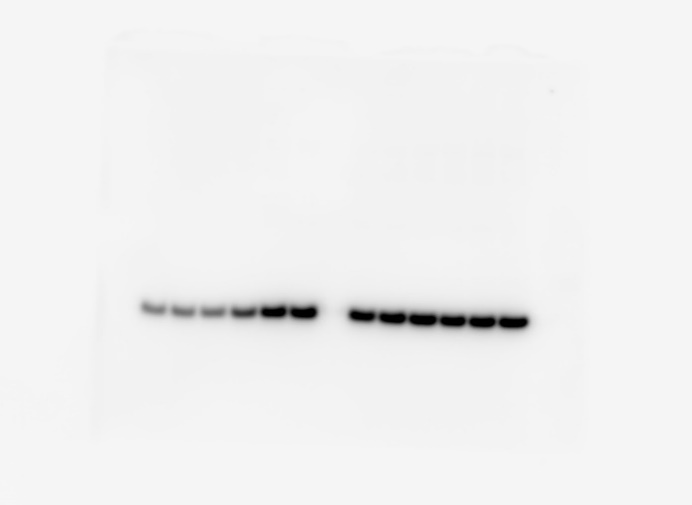

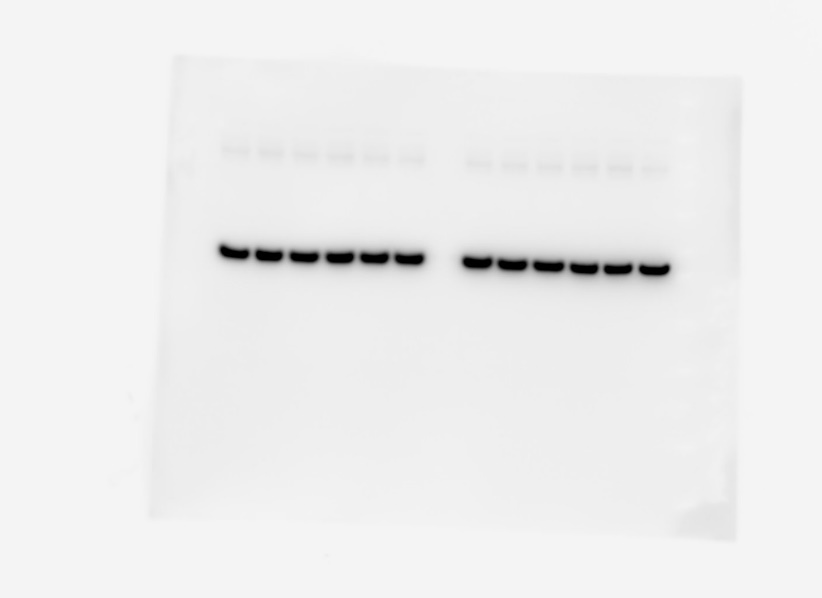

Supplement: Supplementary file 1 — Supplementary Information 1. [file 41598_2023_28846_MOESM1_ESM.docx]
