# Supplementary material for: Mechanisms of amyloid-β34 generation indicate a pivotal role for BACE1 in amyloid homeostasis
Source: Sci Rep. 2023 Feb 7;13:2216. doi: 10.1038/s41598-023-28846-z (PMC9905473; doi:10.1038/s41598-023-28846-z)
Supplement: Supplementary file 2 — Supplementary Information 2. [file 41598_2023_28846_MOESM2_ESM.docx]

**Supplementary Figures and Tables**


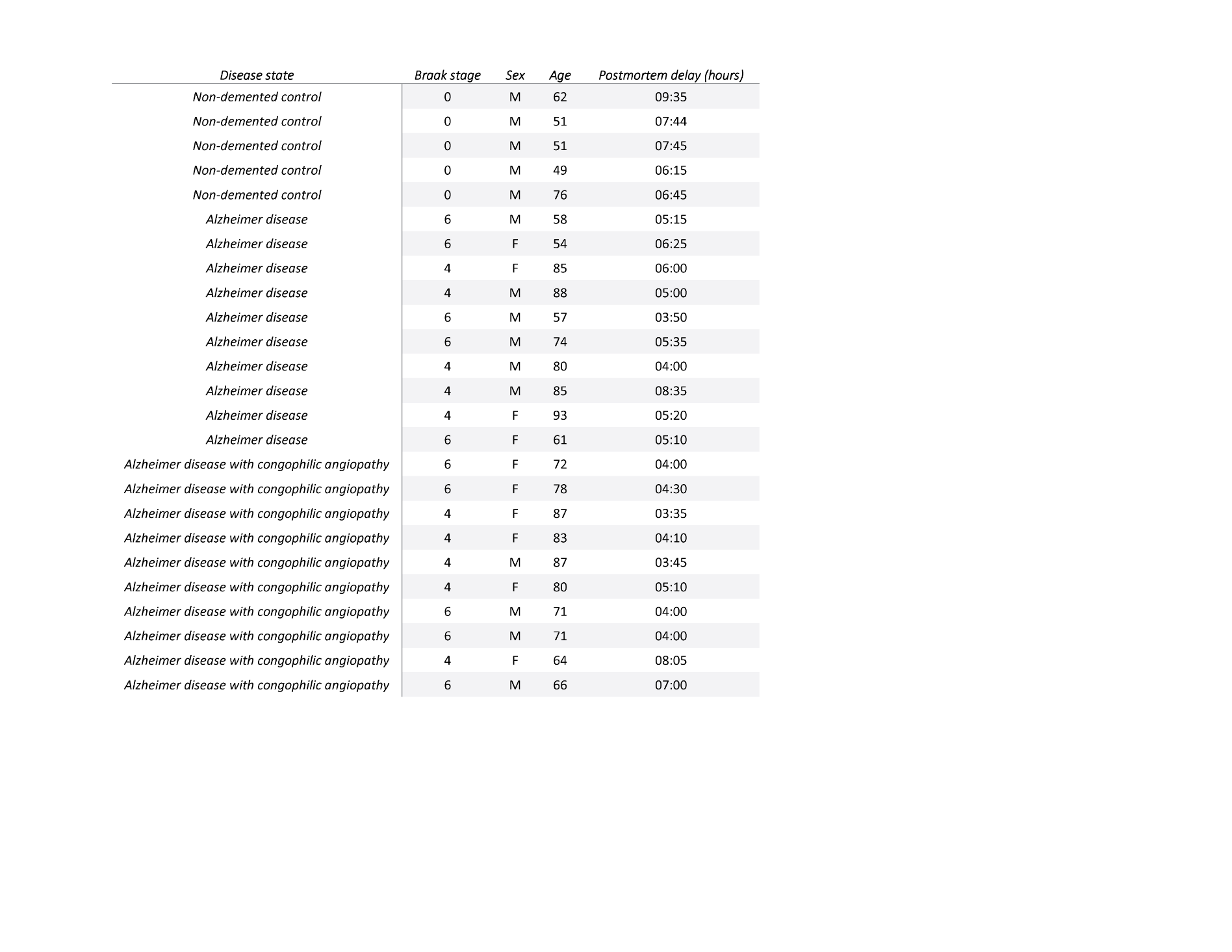
**Supplementary Table 1.** **Disease state, sex and age of the AD patients and controls**

Disease state, Braak stage, sex, age, and postmortem delay of post-mortem brain temporal cortex tissues used for analysis shown in figure 2.


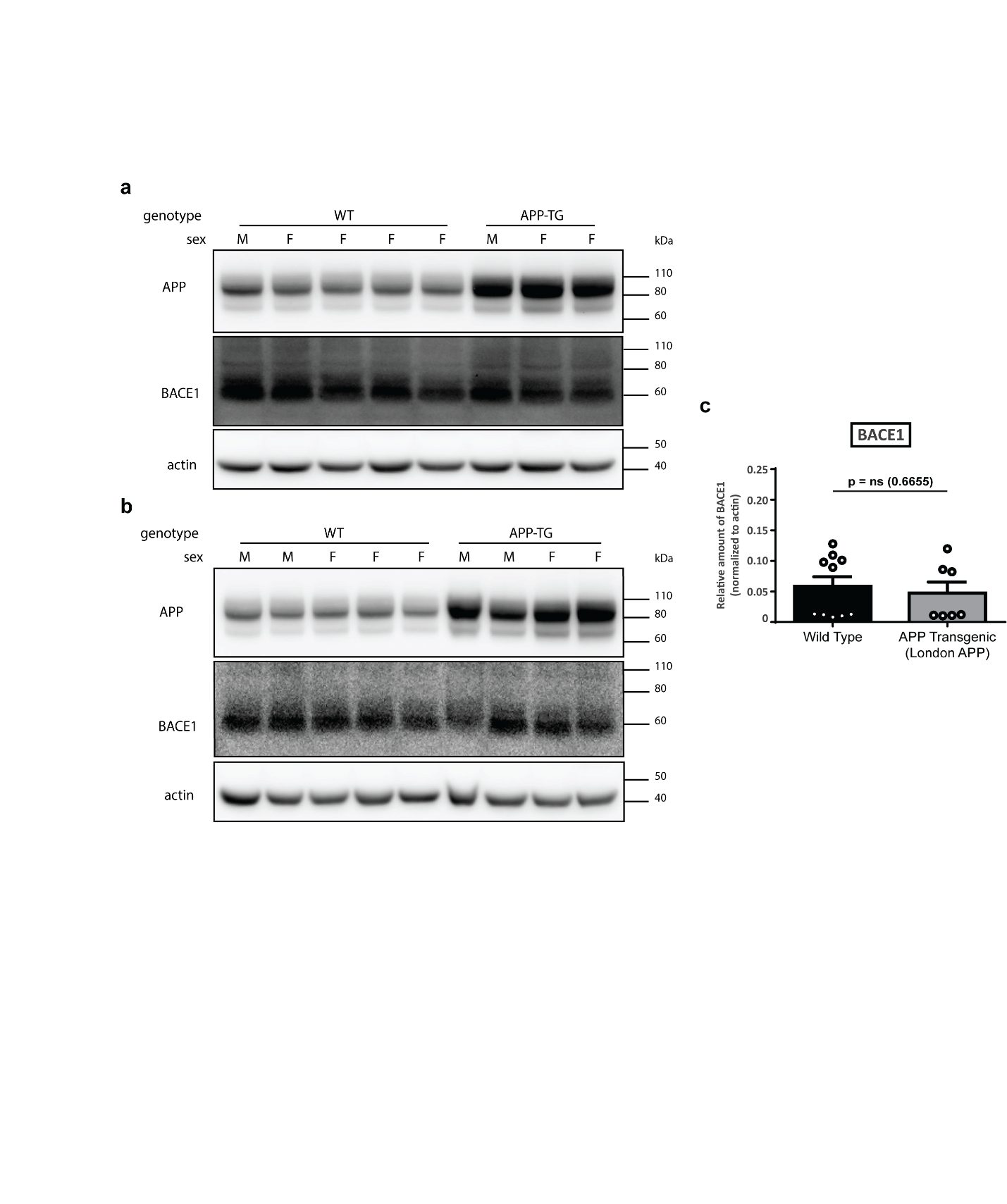


**Supplementary Figure 1. Relative BACE1 levels in cortices of wild type and London APP Transgenic mice**

Expression of BACE1 and APP were analyzed by Western blot (a and b). Western blot quantification of relative amounts of BACE1 (c). Bars and error bars indicate mean ± s.e.m. Unpaired t-test was performed, p = 0.6655.

**
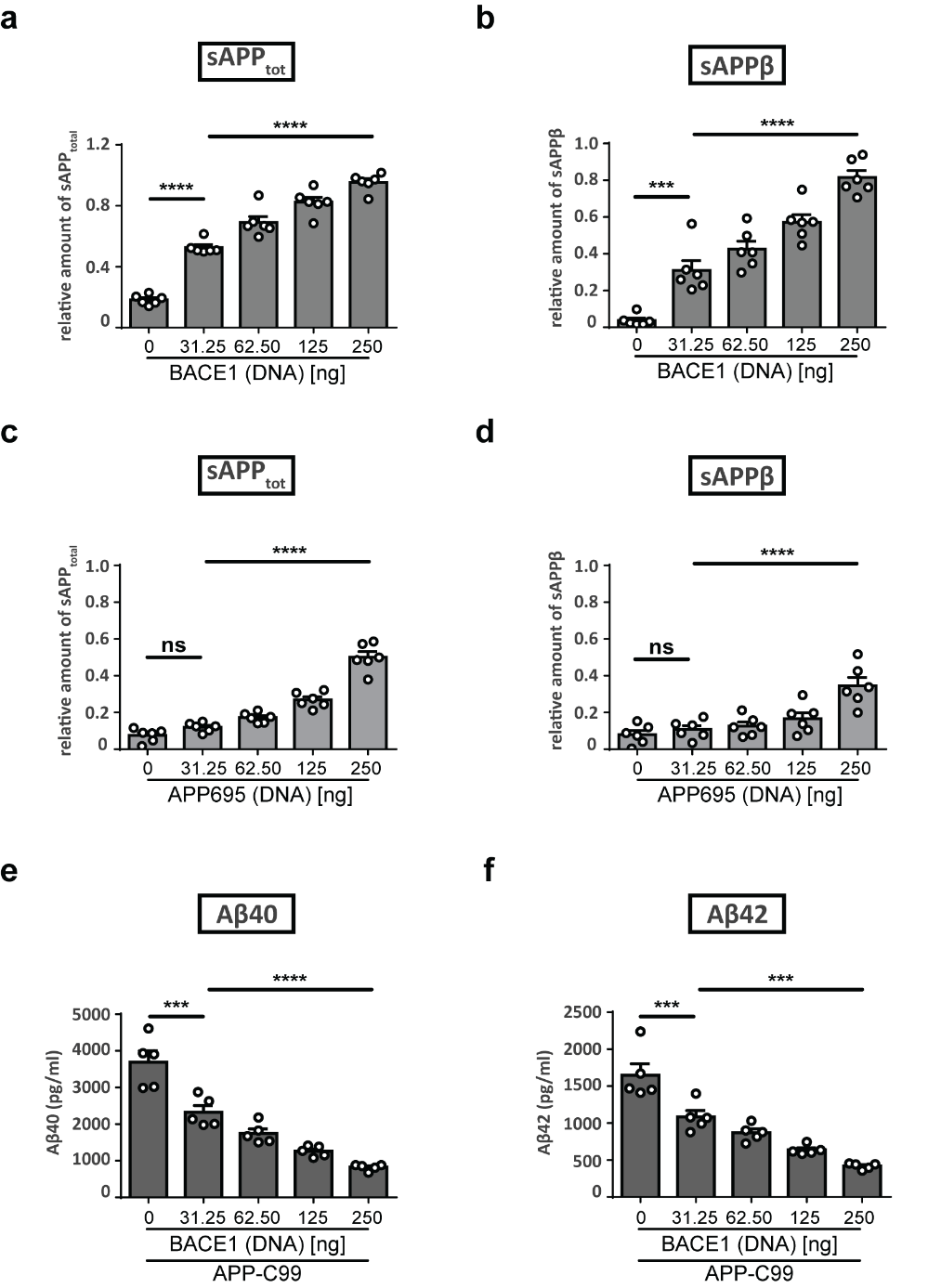
**

**Supplementary Figure 2. APP processing under BACE1, APP695 or APP-C99 overexpression conditions**

Western blot quantification of relative amounts of sAPP_total_ and sAPPβ under BACE1 (a and b) or APP695 (c and d) overexpression conditions presented in Figure 3a and Figure 3b. ELISA quantification of absolute amounts of Aβ40 (e) and Aβ42 (f) under BACE1 and APP-C99 co-overexpression from the experiment presented in Figure 3d. Bars and error bars indicate mean ± s.e.m. Tukey’s post-hoc tests were performed for pairwise comparisons; selected comparisons are highlighted ****p < 0.0001, ***p < 0.001, ns = not significant. (a) sAPP_total_, 1-WAY ANOVA, F(4,25)=124.1, p < 0.0001, (b) sAPPβ, 1-WAY ANOVA, F(4,25)=52.83, p < 0.0001, (c) sAPP_total_, 1-WAY ANOVA, F(4,25)=85.79, p < 0.0001, (d) sAPPβ, 1-WAY ANOVA, F(4,25)=12.35, p < 0.0001, (e) Aβ40, 1-WAY ANOVA, F(4,20)=42.04, p = p < 0.0001, (f) Aβ42, 1-WAY ANOVA, F(4,20)=31.91, p < 0.0001.


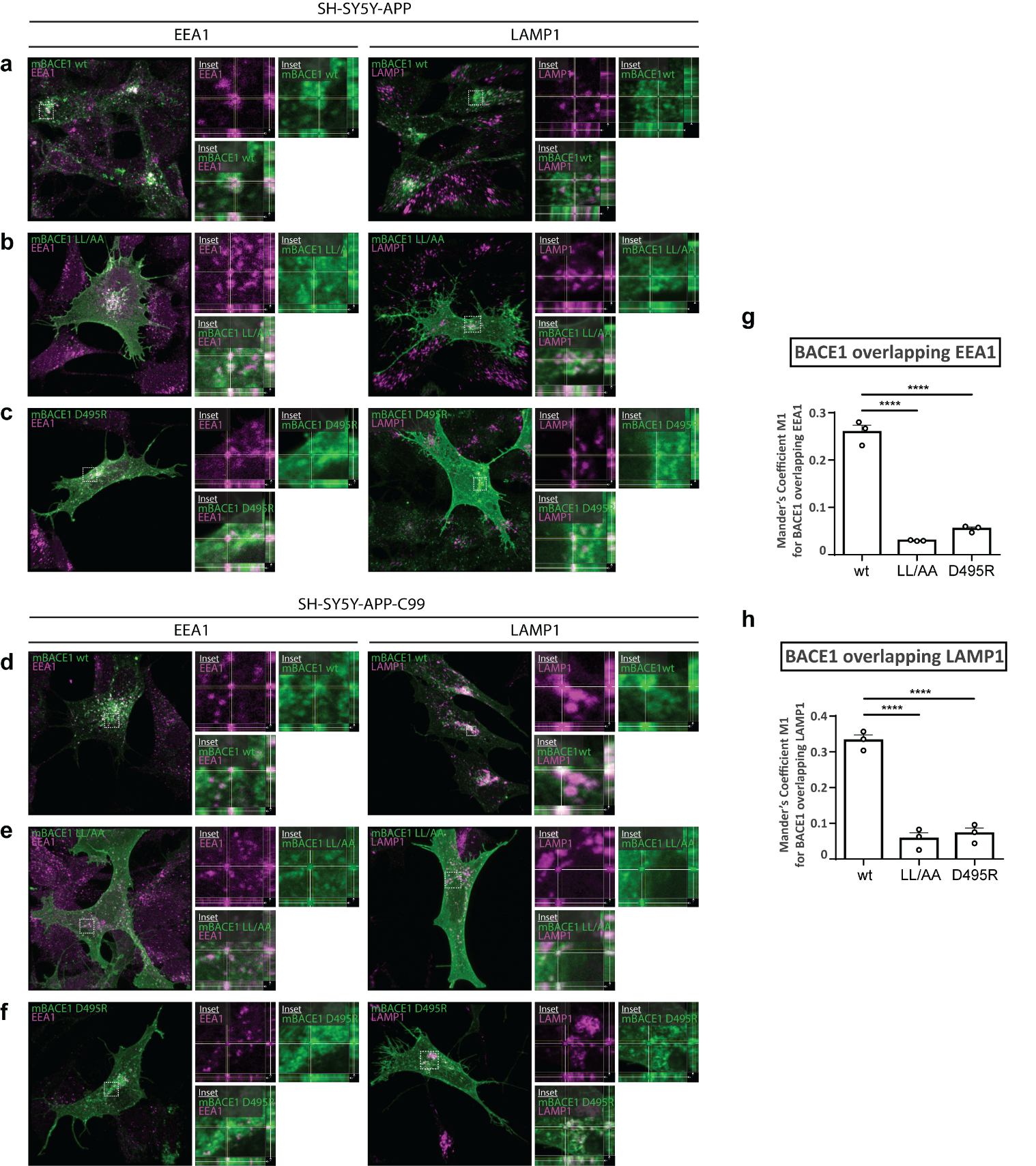


**Supplementary Figure 3. Cellular localization of BACE1 mutants is altered**

Cellular localization of BACE1 mutants was analyzed by ICC from 3 independent experiments with BACE1 transfected SH-SY5H-APP and SH-SY5H-APP-C99 cells. Cross-sectional analysis of co-staining of WT BACE1 (a) and localization mutants of BACE1, D495R (b) and LL/AA (c) with EEA1 and LAMP1 in SH-SY5Y-APP and co-staining of WT BACE1 (d) and localization mutants of BACE1, D495R (e) and LL/AA (f) with EEA1 and LAMP1 in SH-SY5Y-APP-C99 cells. BACE1 colocalization with EEA1 (g) and LAMP1 (h) were analyzed in SH-SY5Y-APP-C99 cells using ImageJ plugin JACoP from 3 independent experiments. Bars and error bars indicate mean ± s.e.m. Dunnett’s post-hoc tests were performed for pairwise comparisons; selected comparisons are highlighted ****p < 0.0001. (g) BACE1 overlapping EEA1, 1-WAY ANOVA, F(2,6)=204.6, p < 0.0001, (h) BACE1 overlapping LAMP1, F(2,6)=96.29, p < 0.0001.


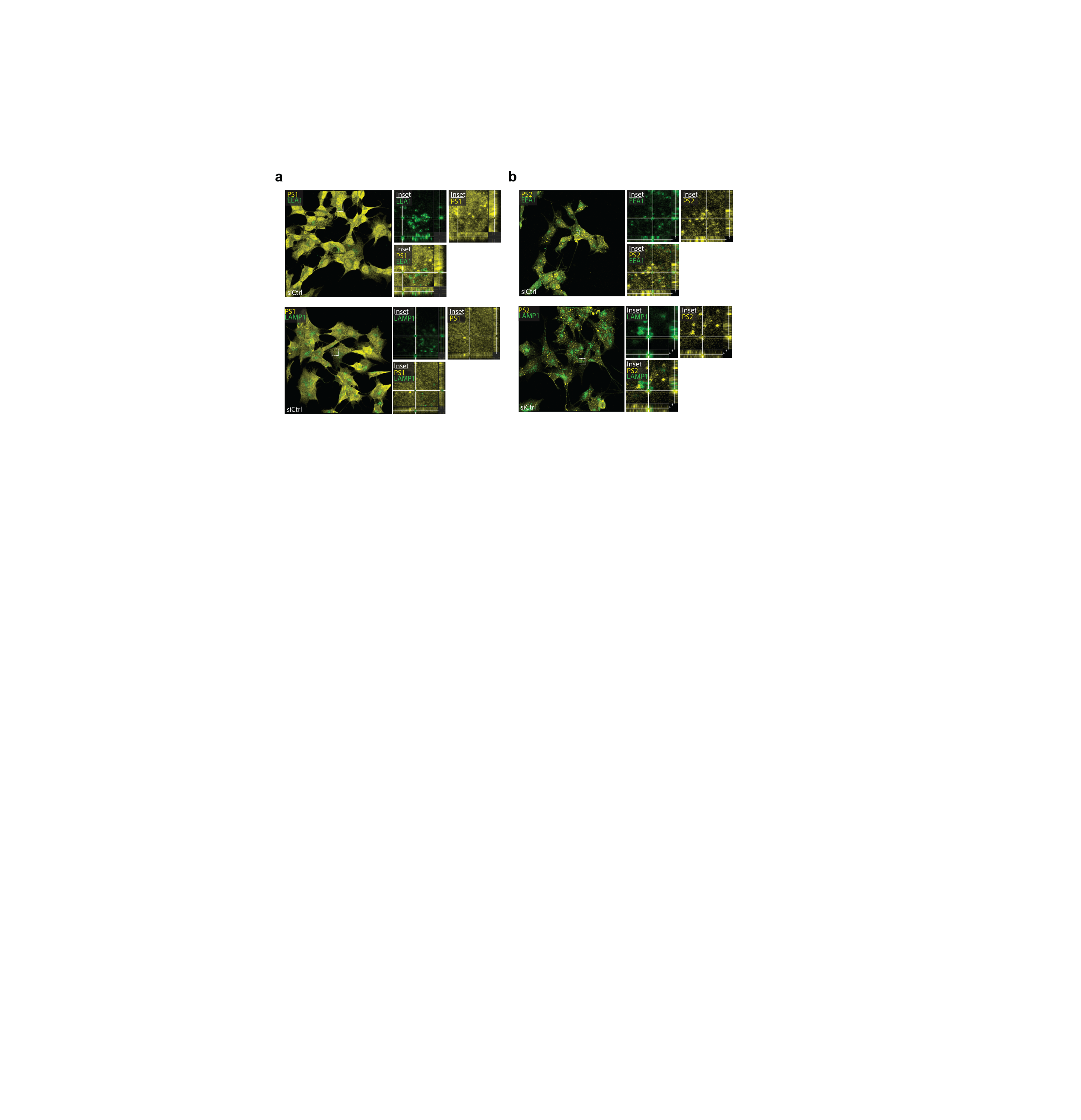


**Supplementary Figure 4. ICC analysis of PS1 and PS2 knockdowns in stably BACE1 overexpressing SH-SY5Y cells**

Relative expression and cellular localization of PS1 and PS2 were analyzed by ICC. ICC analysis from 3 independent experiments for PS1 and PS2 knockdowns in SH-SY5Y-BACE1 cells. Representative cross-sectional analysis of co-staining of PS1 (a) and PS2 (b) with EEA1 and LAMP1.


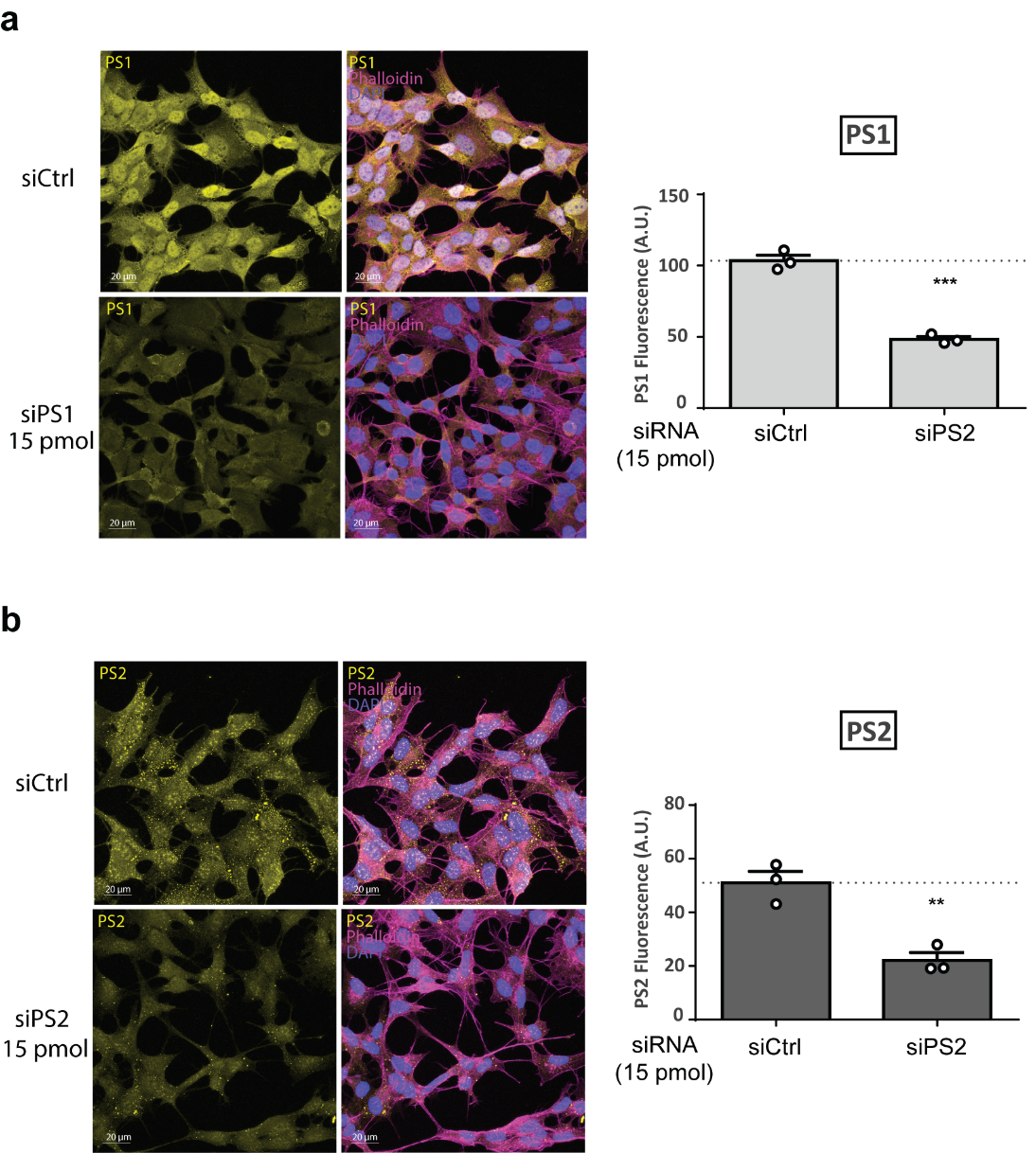


**Supplementary Figure 5. ICC analysis of PS1 and PS2 upon PS1 or PS2 knockdown in stably BACE1 overexpressing SH-SY5Y cells**

Localization of PS1 and PS2 were analyzed by ICC. Representative ICC staining and ICC quantification of PS1 (a) upon PS1 knockdown and PS2 (b) upon PS2 knockdown from 3 independent experiments. Bars and error bars indicate mean ± s.e.m. Unpaired t-test was performed; selected comparisons are highlighted ***p < 0.001, **p < 0.01.

**
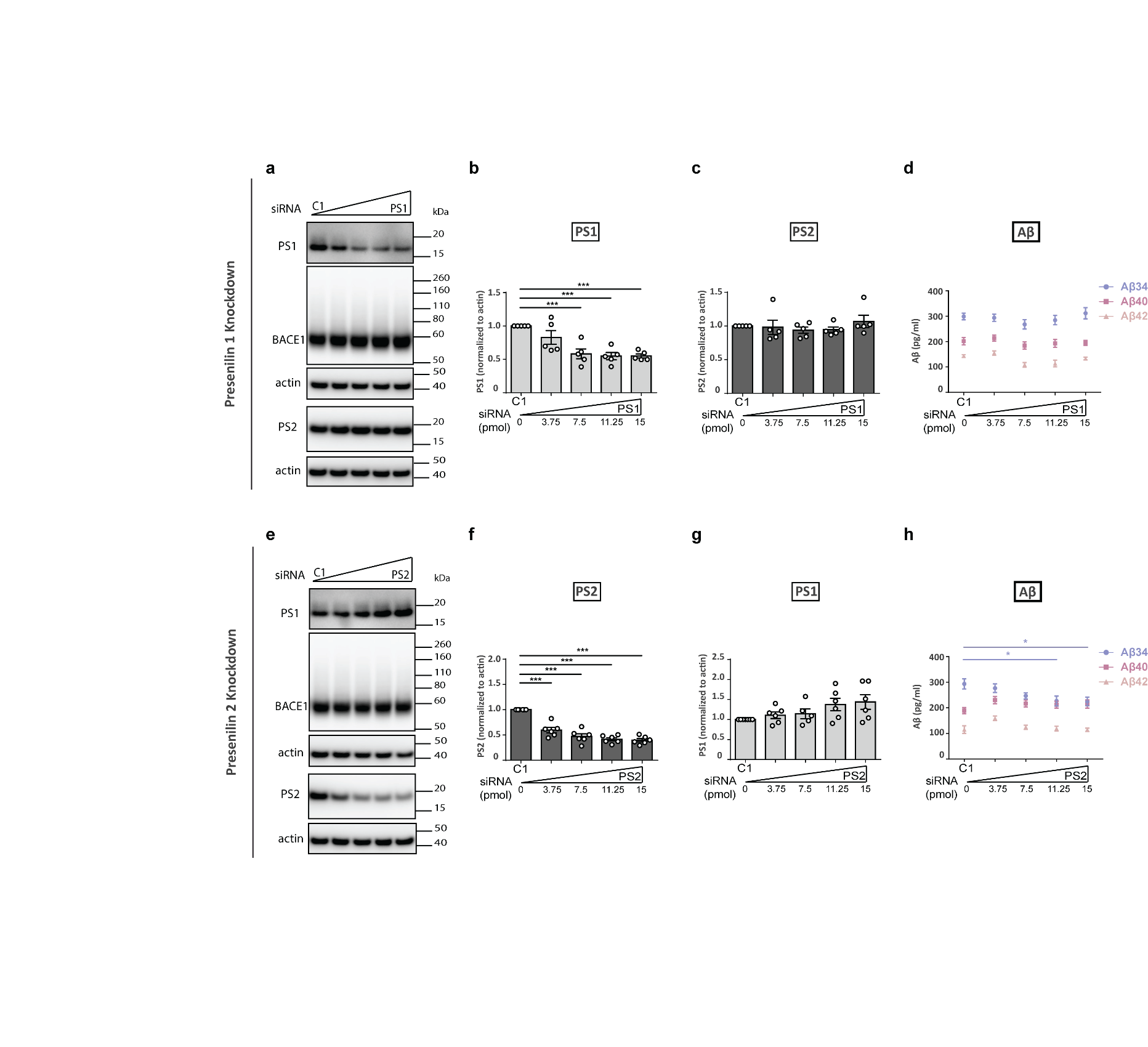
**

**Supplementary Figure 6. Unlike PS1, PS2 knockdown leads to reduced Aβ34 levels in BACE1 overexpressing cells**

Expression of PS1 and PS2 were analyzed by Western blot and Aβ levels determined by ELISA. Data in a-d represent PS1 knockdown and data in e-i represent PS2 knockdown in SH-SY5Y-BACE1 cells. Representative Western blots from 6 independent experiments for PS1 knockdown (a) and from 5 independent experiments for PS2 knockdown (e). Uncropped blots are included in a Supplementary Information file. Western Blot quantification of relative amounts of PS1 (b and g) and of PS2 (c and f), and absolute amounts of Aβ34, Aβ40, and Aβ42 (d and i). Bars and error bars indicate mean ± s.e.m. Dunnett’s post-hoc tests were performed for pairwise comparisons; selected comparisons are highlighted ***p < 0.001, **p < 0.01, *p < 0.05. (b) PS1, 1-WAY ANOVA, F(4,20)=10.33, p < 0.0001, (c) PS2, 1-WAY ANOVA, F(4,20)=0.5776, p = 0.5776, (d) Aβ34, 1-WAY ANOVA, F(4,20)=0.8744, p = 0.4966, Aβ40, 1-WAY ANOVA, F(4,20)=0.6853, p = 0.6104, Aβ42, 1-WAY ANOVA, F(4,20)=4.961, p = 0.0061, (f) PS2, 1-WAY ANOVA, F(4,25)=40.91, p < 0.0001, (g) PS1, 1-WAY ANOVA, F(4,25)=2.448, p = 0.0726, (i) Aβ34, 1-WAY ANOVA, F(4,24)=3.077, p < 0.05, Aβ40, 1-WAY ANOVA, F(4,25)=1.175, p = 0.3456, Aβ42, 1-WAY ANOVA, F(4,24)=3.177, p < 0.05.
